# Adaptive Reliability-Calibrated Consensus–Complementarity–Conflict Modeling for Infrared and Visible Image Fusion

**DOI:** 10.3390/s26154745

**Published:** 2026-07-26

**Authors:** Bowen Tian, Jihao Luo, Ke Lin, Changqing Zhang, Tong Qin

**Affiliations:** School of Information and Electronics, Beijing Institute of Technology, Beijing 100081, China; bowentian_bit@bit.edu.cn (B.T.); jihaoluo_bit@bit.edu.cn (J.L.); 3220241068@bit.edu.cn (K.L.); 3220240889@bit.edu.cn (C.Z.)

**Keywords:** infrared and visible image fusion, multimodal remote sensing, complex-condition perception, trustworthy complementarity verification, conflict-aware routing

## Abstract

Infrared and visible image fusion needs to preserve visible texture details and infrared thermal saliency, yet emphasizing one modality may suppress or distort useful information from the other, while cross-modal differences may also contain noise, pseudo-textures, or locally incompatible boundaries. We propose ARC^3^Fusion, which reformulates image fusion as a reliability-calibrated consensus–complementarity–conflict process to achieve a more effective balance between visible texture detail and infrared target saliency. A progressive shared encoder and a modality-specific residual adapter first produce comparable yet modality-aware features. Cross-Modal Explainable Residual Decomposition then estimates jointly supported consensus and represents the information unexplained by the opposite modality as candidate residuals. Trustworthy Complementarity Verification evaluates infrared residuals using source intensity and edge evidence, while visible residuals are examined using source cues and learnable frequency-pattern evidence. Cross-Modal Conflict Estimation further characterizes local incompatibility through co-activation, reliability, amplitude imbalance, edge-strength mismatch, and orientation mismatch. Conflict-Aware Routing finally coordinates consensus and verified residuals according to these relation cues. Unlike conventional shared–private decomposition that directly preserves private features, ARC^3^Fusion treats modality-specific residuals as candidates that must be verified and conflict-coordinated before fusion. Experiments on LLVIP, MSRS, and TNO demonstrate consistent fusion performance. On LLVIP, ARC^3^Fusion achieves the best EN, SF, AG, VIF, and SCD values of 7.158, 14.467, 4.331, 1.136, and 1.229, respectively. These results indicate that verifying modality-specific residuals and coordinating local conflicts improves the joint preservation of visible texture details and infrared thermal saliency.

## 1. Introduction

Infrared and visible image fusion (IVIF) is an important multimodal sensing technique for autonomous driving, unmanned aerial vehicles, intelligent surveillance, and remote sensing under low illumination, smoke, fog, and other challenging conditions [1,2,3,4,5,6,7]. Infrared images emphasize thermal radiation and salient targets, whereas visible images provide reflectance-dependent textures, edges, colors, and scene details [8,9]. Their fusion is expected to retain both target saliency and structural context so that the resulting representation is more informative for visual interpretation and downstream perception [10,11,12,13].

Existing IVIF methods can be understood from three main perspectives. Source-preservation methods, including encoder–decoder, adversarial, and reconstruction-based designs, aim to retain as much information as possible from both inputs [14,15,16,17,18,19]. Relation-enhancement methods improve feature decomposition, cross-modal interaction, illumination perception, multi-level fusion, or long-range dependency modeling [20,21,22,23,24]. Task-driven and semantic-guided methods further introduce high-level priors or downstream objectives [25,26,27,28]. These directions have substantially improved fusion quality, but they usually optimize how much source information is preserved or how strongly the modalities interact. They less often examine whether a modality-specific difference is reliable and whether two individually useful responses remain structurally compatible at the same location.

This limitation leads to two distinct problems. First, a cross-modal difference is only a candidate for complementarity. Visible high-frequency responses may correspond to useful textures and edges, but may also arise from noise, pseudo-textures, or degradation. Infrared responses may indicate thermal targets, but can also contain diffusion effects, weak boundary perturbations, or sensor noise. Directly injecting such unverified differences can contaminate textures, distort intensities, or blur object boundaries [29,30,31,32,33]. Second, reliable modality-specific information may still conflict locally. Infrared target boundaries and visible texture edges can both be valid while disagreeing in spatial position, gradient strength, or orientation. Related misaligned-fusion studies show that untreated spatial and cross-modal discrepancies can produce ghosting and structural inconsistency [34,35], while semantic- and task-guided studies suggest that higher-level evidence can help constrain which source information should be emphasized [36,37]. These observations motivate the hypothesis of this work: modality differences should be separated from shared structure, verified before preservation, and coordinated when reliable responses compete locally.

For terminology clarity, complementarity in this paper denotes the modality-specific information that is not sufficiently explained by the jointly supported consensus. A candidate residual is the tensor produced before reliability verification and may contain either useful information or disturbances. A trustworthy complementary feature is the reliability-weighted residual retained after verification. The term cue is reserved for observable evidence, such as source intensity, frequency response, edge strength, or gradient orientation, that is used to estimate reliability or conflict.

Based on the above hypothesis, we propose ARC^3^Fusion, an Adaptive Reliability-Calibrated Consensus–Complementarity–Conflict Fusion Network. The method first constructs comparable yet modality-aware features and estimates a soft consensus support between the two modalities. Information that cannot be explained across modalities is represented as candidate residuals rather than immediately treated as useful private information. Source-aware and frequency-domain evidence is then used to verify residual reliability. Finally, local structural conflict is estimated and used to route consensus, infrared-dominant saliency, and visible-domain details.

This process differs technically from conventional shared–private feature decomposition. Shared–private methods generally learn common and modality-specific subspaces and then preserve or combine the private components as useful information [19,20]. In ARC^3^Fusion, modality-specific components are instead defined as consensus-conditioned cross-modal explanation errors. They remain candidates until source and cross-modal evidence confirms their reliability, and even verified residuals are not directly summed; they are coordinated with the consensus through conflict-aware routing. The novelty, therefore, lies not in simply introducing another shared–private split, but in constructing a sequential decision path from consensus estimation to residual verification and conflict-conditioned integration.

The main contributions of this paper are summarized as follows:

- We propose ARC^3^Fusion, a reliability-calibrated IVIF network that formulates fusion as end-to-end consensus–complementarity–conflict relationship modeling instead of direct feature aggregation.
- We design Cross-Modal Explainable Residual Decomposition and Trustworthy Complementarity Verification. Cross-modal differences are first represented as candidate residuals and are then verified using source cues, frequency patterns, and consensus references, reducing the risk of preserving noise-like or degradation-induced responses.
- We introduce Cross-Modal Conflict Estimation and Conflict-Aware Routing to characterize local structural inconsistency and adaptively arbitrate among consensus, infrared saliency, and visible details.
- We introduce auxiliary relationship constraints to regularize consensus decomposition, residual preservation, consensus–residual decorrelation, and frequency-pattern diversity so that the intermediate representations maintain their intended semantic roles.
- Experiments on LLVIP, MSRS, and TNO demonstrate favorable qualitative and quantitative performance, while ablation, relation-map, and pixel-intensity analyses provide complementary evidence for the contributions and intended behavior of the proposed components.

The rest of this paper is organized as follows. Section 2 presents the progressive shared encoder, reliability-calibrated C^3^ fusion strategy, decoder, and optimization objective. Section 3 reports the experimental settings, comparative evaluations, ablation studies, relation-map visualization, pixel-intensity analysis, and computational analysis.

## 2. Methodology

### 2.1. Overview

**Figure 1:**
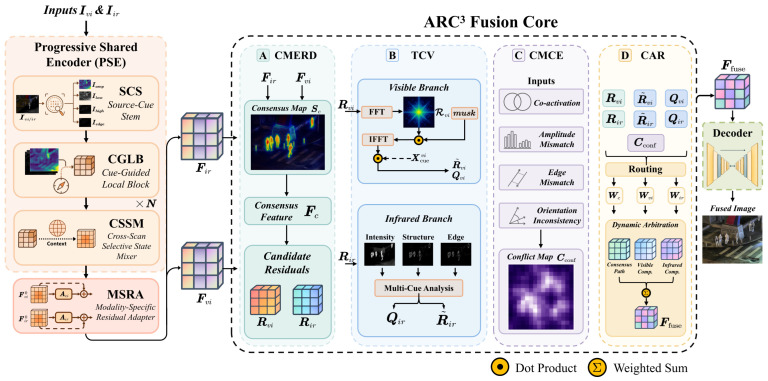
Frameworkof the proposed ARC^3^Fusion. Colors distinguish the major functional modules and feature streams, while the yellow circular symbols denote the operations defined in the legend.

As shown in Figure 1, ARC^3^Fusion follows a progressive encoding–fusing–decoding pipeline. The input infrared and visible images are first processed by the Progressive Shared Encoder (PSE) and the Modality-Specific Residual Adapter (MSRA) to obtain comparable yet modality-aware features. They are then fed into the ARC^3^Fusion Core for consensus anchoring, trustworthy complementarity verification, conflict estimation, and conflict-aware routing. Finally, the decoder reconstructs the fused luminance image from the relation-aware feature. A concise summary of the functions, necessity, and design limitations of the principal components is provided in Appendix A.

### 2.2. Progressive Shared Encoder

To support the subsequent C^3^ fusion strategy, the encoder needs to preserve cross-modal comparability for relation modeling while retaining modality-discriminative cues for complementary information extraction. Specifically, PSE consists of a Source-Cue Stem (SCS), Cue-Guided Local Blocks (CGLBs), and a Cross-Scan Selective State Mixer (CSSM). It is noted that in the PSE, the infrared and visible modalities are processed in parallel using the same layers and shared weights, which helps maintain a comparable feature space for subsequent cross-modal relation modeling. For simplicity, the formulations in this section are described using a generic modality and omit separate infrared and visible subscripts, while the same operations are applied to both modalities in parallel.

#### 2.2.1. Source-Cue Stem

To retain explicit structural priors from the source images at the early encoding stage, we first construct source-cue features for each input modality. Given an input image $I\in R^{1\times H\times W}$, SCS decomposes it into low-frequency, high-frequency, and edge-related responses, and combines them with the original intensity map:

(1) $$I_{\mathrm{low}}=\mathrm{AvgPool}(I),\;\;\;I_{\mathrm{high}}=\left|I-I_{\mathrm{low}}\right|,\;\;\;I_{\mathrm{edge}}=\tanh(\mathrm{Sobel}(I)),$$

(2) $$X_{\mathrm{cue}}=\Phi_{\mathrm{stem}}\left(\left[I,I_{\mathrm{low}},I_{\mathrm{high}},I_{\mathrm{edge}}\right]\right),$$

where *H* and *W* denote the image height and width, respectively, and [·] denotes channel-wise concatenation. Here, Sobel(·) denotes the conventional Sobel gradient operator [38]. The low-frequency component $I_{\mathrm{low}}$ provides smooth illumination and background structure priors, while the residual component $I_{\mathrm{high}}$ highlights local texture variations and possible high-frequency disturbances. In addition, $I_{\mathrm{edge}}$ supplies explicit boundary cues, with tanh(·) used to stabilize the edge response. These complementary cues are fused by the shallow mapping function $\Phi_{\mathrm{stem}}\left(\cdot \right)$, which consists of a standard 3×3 convolution and a depthwise separable convolution [39]. As a result, $X_{\mathrm{cue}}$ provides source-aware structural guidance for subsequent cue-guided feature extraction. This procedure is applied to each modality independently before the shared encoding stages.

#### 2.2.2. Cue-Guided Local Block

**Figure 2:**
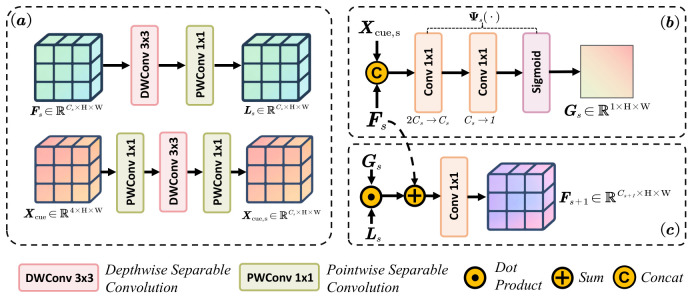
Framework of the proposed Cue-Guided Local Block: (**a**) local-response and source-cue extraction; (**b**) spatial calibration-gate prediction; and (**c**) gated residual modulation and channel expansion. Colors distinguish feature tensors and operation types.

After obtaining the source-cue features, the encoder progressively extracts local responses and calibrates them with shallow structural guidance, as shown in Figure 2. Given the input feature $F_{s}$ at the *s*-th encoding stage, CGLB first derives a local response from the current feature and, meanwhile, projects the source cue into the same stage-specific feature space:

(3) $$L_{s}=\Phi_{\mathrm{loc},s}\left(F_{s}\right),\;\;\;X_{\mathrm{cue},s}=\Phi_{\mathrm{cue},s}\left(X_{\mathrm{cue}}\right),$$

(4) $$\Phi_{\mathrm{loc},s}\left(\cdot \right)={\mathrm{PWConv}}_{1\times 1}^{\mathrm{loc}}\left({\mathrm{DWConv}}_{3\times 3}^{\mathrm{loc}}\left(\cdot \right)\right),$$

(5) $$\Phi_{\mathrm{cue},s}\left(\cdot \right)={\mathrm{PWConv}}_{1\times 1}^{\mathrm{cue},\mathrm{out}}\left({\mathrm{DWConv}}_{3\times 3}^{\mathrm{cue}}\left({\mathrm{PWConv}}_{1\times 1}^{\mathrm{cue},\mathrm{in}}\left(\cdot \right)\right)\right),$$

where $F_{s}$, $L_{s}$, and $X_{\mathrm{cue},s}$ are in $R^{C_{s}\times H\times W}$, denoting the input feature, extracted local response, and stage-wise source-cue feature, respectively. $C_{s}$ is the channel number of the *s*-th stage. The local branch $\Phi_{\mathrm{loc},s}\left(\cdot \right)$ captures lightweight spatial responses from the current feature, where the depthwise convolution focuses on local structures and the pointwise convolution integrates channel-wise responses. In contrast, $\Phi_{\mathrm{cue},s}\left(\cdot \right)$ serves as a source-cue projection branch: the first pointwise convolution aligns the cue with the current stage, the depthwise convolution refines local cue patterns, and the second pointwise convolution integrates the filtered cue responses. In this way, CGLB prepares both feature-domain local responses and source-aware structural guidance for subsequent cue-based calibration.

Since local responses under low-illumination conditions may contain noise-like components, unstable details, and degradation-induced pseudo-high-frequency artifacts, CGLB does not directly inject $L_{s}$ into the main branch. Instead, it predicts a spatial calibration gate from the current feature and the stage-wise source cue:

(6) $$G_{s}=\sigma \left(\Psi_{s}\left(\left[F_{s},X_{\mathrm{cue},s}\right]\right)\right),$$

(7) $${\hat{F}}_{s}=F_{s}+\lambda_{s}G_{s}⊙L_{s},$$

where σ(·) denotes the Sigmoid function, $\Psi_{s}\left(\cdot \right)$ is a lightweight gate prediction function, $G_{s}\in R^{1\times H\times W}$ denotes the spatial gate, ⊙ denotes element-wise multiplication, and $\lambda_{s}$ is a learnable scaling factor. The spatial gate $G_{s}$ is broadcast along the channel dimension when modulating $L_{s}$. ${\hat{F}}_{s}$ denotes the stage output after source-cue modulation. The spatial gate enhances local structures that are consistent with source intensity, frequency, and edge cues, while suppressing noise-like or pseudo-high-frequency responses without sufficient structural support.

Between adjacent CGLBs, we adopt a progressive channel expansion strategy through a 1×1 convolution:

(8) $$F_{s+1}={\mathrm{Conv}}_{1\times 1}\left({\hat{F}}_{s}\right).$$

This strategy gradually increases the representation capacity of the encoder rather than directly projecting shallow features into a high-dimensional space. After multiple CGLB stages, the obtained bottleneck feature $F_{b}$ contains both progressively encoded local structures and effective responses calibrated by explicit source cues.

#### 2.2.3. Cross-Scan Selective State Mixer

**Figure 3:**
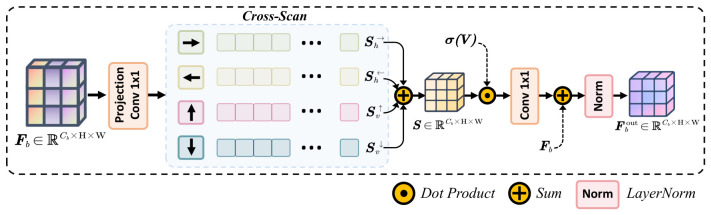
Framework of the proposed Cross-Scan Selective State Mixer. Colors distinguish the different scanning directions, feature tensors, and operation types.

To further introduce broader intra-modal spatial context, we employ the CSSM at the bottleneck of the encoder, as shown in Figure 3. Since local convolutional responses are often insufficient to capture long-range structural continuity, recent studies have explored efficient directional context modeling strategies, such as criss-cross attention, axial attention, and visual selective scanning [40,41,42]. Inspired by these designs, CSSM performs selective state accumulation along horizontal and vertical directions within each modality, rather than prematurely mixing infrared and visible features. This allows each modality to first enhance its own spatial consistency, providing a more stable feature basis for subsequent cross-modal relationship modeling.

Given the bottleneck feature $F_{b}\in R^{C_{b}\times H\times W}$, CSSM first generates the content feature *U*, modulation feature *V*, and selective propagation gate *G*:

(9) $$\left[U,V\right]=\mathrm{Split}\left({\mathrm{Conv}}_{1\times 1}^{2C_{b}}\left(F_{b}\right)\right),\;\;\;G=\sigma \left(\Psi_{g}\left(U\right)\right),$$

where $C_{b}$ denotes the number of bottleneck channels, and $U,V\in R^{C_{b}\times H\times W}$ are obtained by an equal channel-wise split. Here, *U* provides the content to be accumulated along spatial directions, while $G\in {\left[0,\;1\right]}^{C_{b}\times H\times W}$ controls the position- and channel-wise contribution during selective propagation. The modulation feature *V* is retained to further reweight the aggregated contextual state.

Taking the horizontal direction as an example, the forward and backward selective states are defined as follows:

(10) $$S_{h,t}^{\to }=\frac{\sum_{\tau \leq t}G_{\tau }⊙U_{\tau }}{\sum_{\tau \leq t}G_{\tau }+\epsilon },\;\;\;S_{h,t}^{\leftarrow }=\frac{\sum_{\tau \geq t}G_{\tau }⊙U_{\tau }}{\sum_{\tau \geq t}G_{\tau }+\epsilon },$$

(11) $$S_{h}^{\to }={\left\{S_{h,t}^{\to }\right\}}_{t=1}^{W},\;\;\;S_{h}^{\leftarrow }={\left\{S_{h,t}^{\leftarrow }\right\}}_{t=1}^{W},\;\;\;S_{v}^{↓}={\left\{S_{v,t}^{↓}\right\}}_{t=1}^{H},\;\;\;S_{v}^{↑}={\left\{S_{v,t}^{↑}\right\}}_{t=1}^{H},$$

where *t* and τ denote spatial indices along the scan direction, and ϵ is a small constant for numerical stability. The horizontal states $S_{h}^{\to }$ and $S_{h}^{\leftarrow }$ capture bidirectional dependencies along each row, while the vertical states $S_{v}^{↓}$ and $S_{v}^{↑}$ provide analogous structural propagation along each column. Afterwards, the bidirectional states from the two scan directions are aggregated as follows:

(12) $$S_{h}=\frac{1}{2}\left(S_{h}^{\to }+S_{h}^{\leftarrow }\right),\;\;\;S_{v}=\frac{1}{2}\left(S_{v}^{↓}+S_{v}^{↑}\right),\;\;\;S=\frac{1}{2}\left(S_{h}+S_{v}\right).$$

Here, $S_{h}$ and $S_{v}$ encode horizontal and vertical structural continuity, respectively, and their fusion produces the cross-directional contextual state *S*. Finally, CSSM uses the modulation feature *V* to select useful contextual responses and injects the enhanced context into the bottleneck feature in a residual form:

(13) $$F_{\mathrm{cssm}}={\mathrm{Conv}}_{1\times 1}\left(S⊙\sigma (V)\right),\;\;\;F_{b}^{\mathrm{out}}=\mathrm{Norm}\left(F_{b}+\lambda_{c}F_{\mathrm{cssm}}\right),$$

where $F_{\mathrm{cssm}}$ denotes the modulated contextual feature, Norm(·) denotes the normalization operation, and $\lambda_{c}$ is a learnable scaling factor. The output $F_{b}^{\mathrm{out}}$, therefore, contains direction-aware contextual information while preserving the original modality-specific responses. Overall, CSSM improves intra-modal contextual consistency through efficient directional state accumulation, providing a more stable feature basis for subsequent cross-modal residual decomposition.

### 2.3. Modality-Specific Residual Adapter

**Figure 4:**
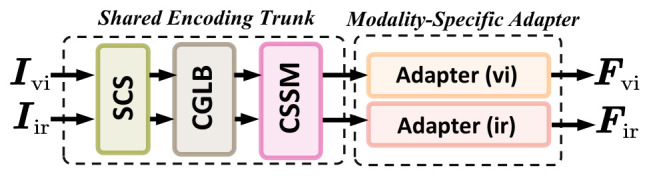
Framework of the shared encoding trunk and the proposed Modality-Specific Residual Adapter.

Although the shared encoding trunk helps maintain cross-modal comparability, excessive parameter sharing may weaken modality-specific responses that are important for infrared–visible fusion. In particular, infrared features usually contain thermal saliency and object-background contrast, while visible features provide richer texture and structural details. To compensate for these modality-dependent cues without breaking the comparable feature space established by the shared trunk, we introduce the MSRA after the shared trunk for lightweight residual calibration, as shown in Figure 4.

Given a visible image $I_{\mathrm{vi}}$ and an infrared image $I_{\mathrm{ir}}$, the shared encoding trunk first extracts the base features:

(14) $$F_{\mathrm{vi}}^{0}={\mathrm{PSE}}_{\mathrm{trunk}}\left(I_{\mathrm{vi}}\right),\;\;\;F_{\mathrm{ir}}^{0}={\mathrm{PSE}}_{\mathrm{trunk}}\left(I_{\mathrm{ir}}\right),$$

where ${\mathrm{PSE}}_{\mathrm{trunk}}\left(\cdot \right)$ denotes the shared encoding trunk before MSRA, and $F_{\mathrm{vi}}^{0}$ and $F_{\mathrm{ir}}^{0}$ denote the visible and infrared base features before modality-specific calibration, respectively. These base features provide a comparable representation for subsequent cross-modal relationship modeling, but they may not fully preserve modality-dependent responses. Therefore, MSRA introduces two lightweight adaptation branches with unshared parameters:

(15) $$F_{\mathrm{vi}}=F_{\mathrm{vi}}^{0}+\eta_{\mathrm{vi}}A_{\mathrm{vi}}\left(F_{\mathrm{vi}}^{0}\right),\;\;\;F_{\mathrm{ir}}=F_{\mathrm{ir}}^{0}+\eta_{\mathrm{ir}}A_{\mathrm{ir}}\left(F_{\mathrm{ir}}^{0}\right),$$

(16) $$A_{m}\left(F\right)={\mathrm{PWConv}}_{1\times 1}^{m}\left({\mathrm{DWConv}}_{3\times 3}^{m}\left(F\right)\right),\;\;\;m\in \left\{\mathrm{vi},\mathrm{ir}\right\},$$

where $A_{\mathrm{vi}}\left(\cdot \right)$ and $A_{\mathrm{ir}}\left(\cdot \right)$ denote the visible and infrared adaptation branches, respectively. The additive feature-calibration form follows the residual learning principle [43], while the two branches share the same lightweight structure but use unshared parameters to capture modality-specific residual responses. $\eta_{\mathrm{vi}}$ and $\eta_{\mathrm{ir}}$ are learnable scaling factors that control the strength of residual calibration.

In this way, MSRA refines the shared features through a residual path rather than replacing them, reducing the risk of disrupting cross-modal comparability. Consequently, the final features $F_{\mathrm{vi}}$ and $F_{\mathrm{ir}}$ preserve a comparable feature basis while retaining visible texture details and infrared thermal responses for subsequent fusion.

### 2.4. ARC^3^Fusion Strategy

The proposed ARC^3^Fusion Strategy formulates infrared and visible feature fusion as a progressive end-to-end process of consensus, complementarity, and conflict modeling. Rather than achieving fusion through sophisticated module stacking, our method emphasizes the explicit semantic roles of intermediate representations and integrates cross-modal explainable residual decomposition, trustworthy complementarity verification, cross-modal conflict estimation, and conflict-aware routing into a unified fusion core, where candidate difference generation, reliability screening, structural conflict estimation, and adaptive feature integration are jointly modeled. Specifically, the ARC^3^Fusion Core consists of Cross-Modal Explainable Residual Decomposition (CMERD), Trustworthy Complementarity Verification (TCV), Cross-Modal Conflict Estimation (CMCE), and Conflict-Aware Routing (CAR).

#### 2.4.1. Cross-Modal Explainable Residual Decomposition

**Figure 5:**
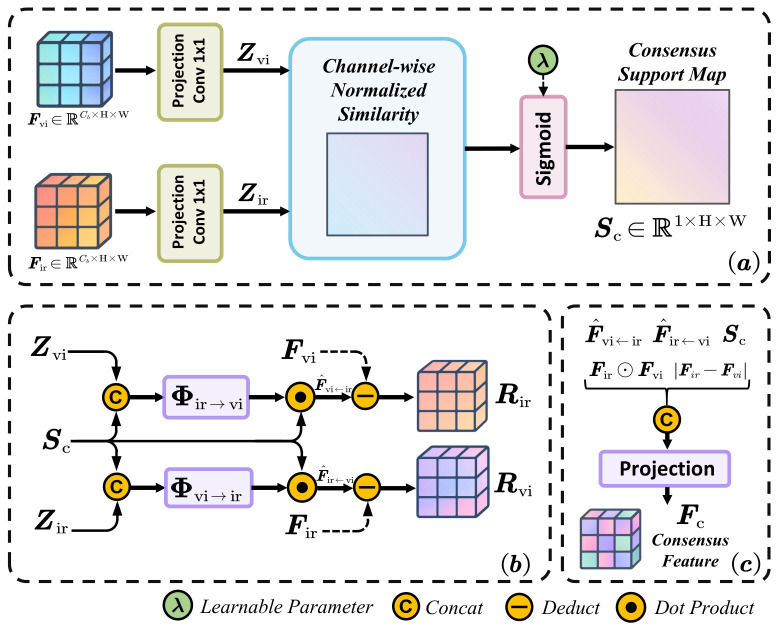
Framework of the proposed Cross-Modal Explainable Residual Decomposition: (**a**) consensus-support estimation; (**b**) bidirectional cross-modal explanation and candidate-residual construction; and (**c**) consensus-feature construction. Colors distinguish the visible, infrared, and consensus feature streams.

In the ARC^3^Fusion core, we first decompose the dual-modal features into two parts: stable structures jointly supported by both modalities and modality-specific responses that cannot be sufficiently explained by the other modality, as shown in Figure 5. This decomposition is important because not all cross-modal differences are reliable complementary information; some of them may originate from noise, degradation, or modality-specific artifacts.

Given infrared and visible features $F_{\mathrm{ir}},F_{\mathrm{vi}}\in R^{C_{b}\times H\times W}$, we first project them into a shared relation-comparison space through lightweight projection functions:

(17) $$Z_{\mathrm{vi}}=P_{\mathrm{vi}}\left(F_{\mathrm{vi}}\right),\;Z_{\mathrm{ir}}=P_{\mathrm{ir}}\left(F_{\mathrm{ir}}\right),$$

where $Z_{\mathrm{vi}},Z_{\mathrm{ir}}\in R^{C_{b}\times H\times W}$ are the projected visible and infrared features, and $C_{b}$ denotes the number of bottleneck feature channels. The projection functions $P_{\mathrm{vi}}\left(\cdot \right)$ and $P_{\mathrm{ir}}\left(\cdot \right)$ align the channel representations of the two modalities, so that their local relations can be compared more stably. Based on the projected features, we compute a normalized channel-wise similarity at each spatial position to estimate the consensus support map:

(18) $$S_{c}=\sigma \left(\gamma \frac{{\langle Z_{\mathrm{vi}},Z_{\mathrm{ir}}\rangle }_{\mathrm{ch}}}{{∥Z_{\mathrm{vi}}∥}_{2,\mathrm{ch}}{∥Z_{\mathrm{ir}}∥}_{2,\mathrm{ch}}+\epsilon }\right),$$

where ${\langle \cdot ,\cdot \rangle }_{\mathrm{ch}}$ and ${∥\cdot ∥}_{2,\mathrm{ch}}$ denote the channel-wise inner product and $L_{2}$ norm, respectively. The normalized inner-product term follows the conventional cosine-similarity formulation [44], while the learnable scaling factor γ and Sigmoid mapping convert it into a spatial consensus-support estimate. Here, γ is constrained to be non-negative, and ϵ is used for numerical stability. The obtained $S_{c}\in {\left[0,1\right]}^{1\times H\times W}$ measures the local common support between the two modalities and serves as a consensus prior. Rather than directly deciding which source feature should be preserved, it provides a soft structural reference for the following explanation and decomposition.

After obtaining the consensus support map, CMERD constructs bidirectional cross-modal explanation mappings to estimate the components in one modality that can be explained by the other modality:

(19) $${\bar{F}}_{\mathrm{ir}\leftarrow \mathrm{vi}}=\Phi_{\mathrm{vi}\to \mathrm{ir}}\left(\left[Z_{\mathrm{vi}},S_{c}\right]\right),\;\;\;{\hat{F}}_{\mathrm{ir}\leftarrow \mathrm{vi}}=S_{c}⊙{\bar{F}}_{\mathrm{ir}\leftarrow \mathrm{vi}},$$

(20) $${\bar{F}}_{\mathrm{vi}\leftarrow \mathrm{ir}}=\Phi_{\mathrm{ir}\to \mathrm{vi}}\left(\left[Z_{\mathrm{ir}},S_{c}\right]\right),\;\;\;{\hat{F}}_{\mathrm{vi}\leftarrow \mathrm{ir}}=S_{c}⊙{\bar{F}}_{\mathrm{vi}\leftarrow \mathrm{ir}},$$

where $\Phi_{\mathrm{vi}\to \mathrm{ir}}\left(\cdot \right)$ and $\Phi_{\mathrm{ir}\to \mathrm{vi}}\left(\cdot \right)$ denote the visible-to-infrared and infrared-to-visible explanation mappings, respectively. The unconstrained predictions ${\bar{F}}_{\mathrm{ir}\leftarrow \mathrm{vi}}$ and ${\bar{F}}_{\mathrm{vi}\leftarrow \mathrm{ir}}$ estimate cross-modal explainable components, while ${\hat{F}}_{\mathrm{ir}\leftarrow \mathrm{vi}}$ and ${\hat{F}}_{\mathrm{vi}\leftarrow \mathrm{ir}}$ are further constrained by the consensus support. In this way, the explanation process is encouraged to focus on regions with strong common support, reducing the risk of forcing cross-modal explanations in structurally inconsistent areas.

Based on these bidirectional explainable components, the infrared and visible candidate residuals are defined as follows:

(21) $$R_{\mathrm{ir}}=F_{\mathrm{ir}}-{\hat{F}}_{\mathrm{ir}\leftarrow \mathrm{vi}},\;R_{\mathrm{vi}}=F_{\mathrm{vi}}-{\hat{F}}_{\mathrm{vi}\leftarrow \mathrm{ir}},$$

where $R_{\mathrm{ir}}$ and $R_{\mathrm{vi}}$ represent modality-specific differences that cannot be sufficiently explained by the other modality. However, these residuals are only candidate complementary responses, rather than directly trustworthy complementary information. Therefore, their reliability is further verified in TCV.

Meanwhile, CMERD also constructs a consensus feature by integrating bidirectional explainable components, original feature relations, and the consensus support the following:

(22) $$X_{c}=[{\hat{F}}_{\mathrm{ir}\leftarrow \mathrm{vi}},{\hat{F}}_{\mathrm{vi}\leftarrow \mathrm{ir}},F_{\mathrm{ir}}⊙F_{\mathrm{vi}},|F_{\mathrm{ir}}-F_{\mathrm{vi}}\left|,S_{c}\right],\;F_{c}=\Phi_{c}\left(X_{c}\right),$$

where $X_{c}$ collects consensus-related evidence from explainable components, feature interaction, feature discrepancy, and common support. The mapping function $\Phi_{c}\left(\cdot \right)$ further converts these cues into the consensus feature $F_{c}$. Since $F_{c}$ is jointly constrained by consensus support and bidirectional explanation, it provides a stable structural anchor for subsequent complementarity verification and conflict-aware routing.

To prevent the explanation mappings from degenerating into unconstrained convolutional transformations, we introduce three auxiliary constraints: regional explanation, residual preservation, and consensus-residual decoupling. First, the regional explanation constraint suppresses residual responses in high-consensus regions:

(23) $$L_{\exp}=E\left(S_{c}⊙R_{\mathrm{ir}}\right)+E\left(S_{c}⊙R_{\mathrm{vi}}\right),\;\;\;E\left(\cdot \right)=\frac{1}{CHW}{∥\cdot ∥}_{1},$$

where E(·) denotes the normalized $L_{1}$ energy measure. This constraint encourages regions with strong consensus support to be explained by the cross-modal consensus branch, rather than being unnecessarily left in the residual branches.

However, overly suppressing residuals may weaken truly modality-specific information. Therefore, we further impose a hinge-based residual preservation constraint on low-consensus regions:

(24) $$L_{\mathrm{res}}=\max\left(0,\rho -E(\left(1-S_{c}\right)⊙R_{\mathrm{ir}})\right)+\max\left(0,\rho -E(\left(1-S_{c}\right)⊙R_{\mathrm{vi}})\right),$$

where ρ denotes the residual preservation threshold. This term provides a lower-bound regularization for residual energy in low-consensus regions, ensuring sufficient representation capacity for modality-specific responses without encouraging unlimited residual amplification.

Furthermore, to reduce redundant overlap between the consensus and residual branches, we introduce a consensus–residual decoupling constraint based on the cosine similarity between flattened feature tensors:

(25) $$L_{\mathrm{orth}}=\max\left(0,|\mathrm{sim}(R_{\mathrm{ir}},F_{c})|-\delta \right)+\max\left(0,|\mathrm{sim}(R_{\mathrm{vi}},F_{c})|-\delta \right),$$

where sim(·,·) denotes the cosine similarity computed after flattening the feature tensors, and δ is a tolerance threshold for allowed correlation. Instead of enforcing strict orthogonality, this constraint only penalizes excessive correlation beyond the threshold. As a result, it reduces redundant information overlap while preserving necessary structural association between consensus and residual representations.

Through CMERD, the dual-modal features are decomposed into the consensus feature $F_{c}$ and candidate residuals $R_{\mathrm{ir}}$ and $R_{\mathrm{vi}}$. These explicitly defined representations provide a clear basis for subsequent trustworthy complementarity verification.

#### 2.4.2. Trustworthy Complementarity Verification

**Figure 6:**
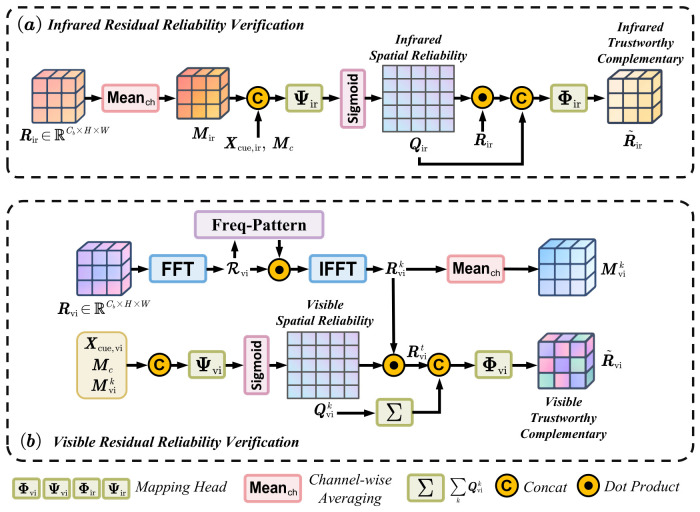
Framework of the proposed Trustworthy Complementarity Verification: (**a**) infrared residual reliability verification; and (**b**) visible residual frequency-pattern verification. Colors distinguish the feature streams and operation types.

After CMERD, the network obtains the consensus feature $F_{c}$ and the candidate residuals $R_{\mathrm{ir}}$ and $R_{\mathrm{vi}}$. However, these residuals only indicate modality differences and may contain both useful complementary information and unreliable responses, such as noise, pseudo-textures, or degradation-induced artifacts. Therefore, we propose the TCV to verify the reliability of infrared and visible residuals before using them for fusion, as shown in Figure 6. Since infrared residuals mainly reflect thermal saliency, while visible residuals usually contain richer texture and frequency-dependent details, TCV adopts modality-aware verification strategies for the two branches.

For the infrared candidate residual, the verification focuses on whether its spatial response is supported by source intensity, thermal structure, and edge cues. Specifically, we compute the residual magnitude map and construct infrared source cues as follows:

(26) $$M_{\mathrm{ir}}={\mathrm{Mean}}_{\mathrm{ch}}\left(\right|R_{\mathrm{ir}}\left|\right),\;I_{\mathrm{ir}}^{\mathrm{low}}=\mathrm{AvgPool}\left(I_{\mathrm{ir}}\right),$$

(27) $$I_{\mathrm{ir}}^{\mathrm{high}}=|I_{\mathrm{ir}}-I_{\mathrm{ir}}^{\mathrm{low}}|,\;E_{\mathrm{ir}}=\tanh\left(\mathrm{Sobel}\left(I_{\mathrm{ir}}\right)\right),\;M_{c}={\mathrm{Mean}}_{\mathrm{ch}}\left(\right|F_{c}\left|\right),$$

where ${\mathrm{Mean}}_{\mathrm{ch}}\left(\cdot \right)$ denotes channel-wise averaging. $M_{\mathrm{ir}}$ describes the spatial saliency of the infrared residual, while $I_{\mathrm{ir}}$, $I_{\mathrm{ir}}^{\mathrm{low}}$, $I_{\mathrm{ir}}^{\mathrm{high}}$, and $E_{\mathrm{ir}}$ provide source intensity, low-frequency, high-frequency, and edge cues, respectively. In addition, $M_{c}$ serves as a consensus structural reference, helping distinguish reliable residual responses from unstable modality-specific disturbances. Based on these cues, the infrared reliability map is predicted as follows:

(28) $$Q_{\mathrm{ir}}=\sigma \left(\Psi_{\mathrm{ir}}\left(\left[M_{\mathrm{ir}},I_{\mathrm{ir}},I_{\mathrm{ir}}^{\mathrm{high}},I_{\mathrm{ir}}^{\mathrm{low}},E_{\mathrm{ir}},M_{c}\right]\right)\right),\;R_{\mathrm{ir}}^{t}=Q_{\mathrm{ir}}⊙R_{\mathrm{ir}},$$

where $\Psi_{\mathrm{ir}}\left(\cdot \right)$ denotes the infrared reliability prediction head, and $Q_{\mathrm{ir}}\in {\left[0,1\right]}^{1\times H\times W}$ measures the spatial reliability of the infrared candidate residual. $Q_{\mathrm{ir}}$ is broadcast along the channel dimension when modulating $R_{\mathrm{ir}}$, producing the reliability-weighted residual $R_{\mathrm{ir}}^{t}$. The weighted residual is then mapped into an infrared trustworthy complementary feature:

(29) $${\tilde{R}}_{\mathrm{ir}}=\Phi_{\mathrm{ir}}\left(\left[R_{\mathrm{ir}}^{t},Q_{\mathrm{ir}}\right]\right),$$

where $\Phi_{\mathrm{ir}}\left(\cdot \right)$ denotes the infrared complementary feature mapping function, and ${\tilde{R}}_{\mathrm{ir}}$ denotes the verified infrared complementary feature. In this way, infrared complementary information is preserved only when its residual response is supported by source-aware structural evidence rather than by magnitude alone.

For the visible candidate residual, the verification needs to further distinguish reliable textures from pseudo-texture artifacts. To this end, we introduce a constrained frequency-pattern verification mechanism. We first apply a channel-wise two-dimensional Fourier transform to $R_{\mathrm{vi}}$ and define the normalized frequency radius:

(30) $$R_{\mathrm{vi}}=F\left(R_{\mathrm{vi}}\right),\;\;\;r\left(u,v\right)=\frac{\sqrt{{\left(\frac{u-u_{0}}{H}\right)}^{2}+{\left(\frac{v-v_{0}}{W}\right)}^{2}}}{r_{\max}},$$

where F(·) denotes the conventional two-dimensional discrete Fourier transform [45], $R_{\mathrm{vi}}$ is the frequency-domain representation of the visible residual, (u,v) denotes the frequency position, and $\left(u_{0},v_{0}\right)$ denotes the spectrum center. The normalized frequency radius r(u,v) and the subsequent learnable frequency-pattern masks are constructed in this work for visible residual verification, where $r_{\max}$ is the maximum value of the unnormalized radius over the frequency plane. Then, *K* radially ordered frequency-pattern masks are constructed as follows:

(31) $$G_{k}\left(u,v\right)=\frac{\exp\left(-\frac{{\left(r\left(u,v\right)-\mu_{k}\right)}^{2}}{2b_{k}^{2}}\right)}{\sum_{j=1}^{K}\exp\left(-\frac{{\left(r\left(u,v\right)-\mu_{j}\right)}^{2}}{2b_{j}^{2}}\right)},\;\;\;\sum_{k=1}^{K}G_{k}\left(u,v\right)=1,$$

where $G_{k}\left(u,v\right)$ denotes the *k*-th frequency-pattern mask, $\mu_{k}$ and $b_{k}$ denote its normalized center frequency and bandwidth, respectively, and *K* is the number of frequency patterns. To maintain the radial order of different frequency patterns, we constrain $0<\mu_{1}<\mu_{2}<\cdots <\mu_{K}<1$. In implementation, we set $d_{j}=\mathrm{softplus}\left(a_{j}\right)$ and use the following increasing parameterization:

(32) $$\mu_{k}=\frac{\sum_{j=1}^{k}d_{j}}{\sum_{j=1}^{K+1}d_{j}},$$

where softplus(·) ensures $d_{j}>0$, and $a_{j}$ is a learnable parameter. With these ordered masks, the visible residual is decomposed into frequency-pattern responses:

(33) Rvik=ReF−1(Gk⊙Rvi),

where $F^{-1}\left(\cdot \right)$ denotes the inverse Fourier transform, Re[·] takes the real part, and $R_{\mathrm{vi}}^{k}$ denotes the *k*-th visible residual frequency pattern. This decomposition allows the visible branch to verify structural variations, texture details, and possible pseudo-high-frequency responses in a more fine-grained manner.

For each frequency pattern, we compute its spatial magnitude and combine it with visible source cues and the consensus reference:

(34) $$M_{\mathrm{vi}}^{k}={\mathrm{Mean}}_{\mathrm{ch}}\left(\right|R_{\mathrm{vi}}^{k}\left|\right),\;\;\;I_{\mathrm{vi}}^{\mathrm{low}}=\mathrm{AvgPool}\left(I_{\mathrm{vi}}\right),$$

(35) $$I_{\mathrm{vi}}^{\mathrm{high}}=\left|I_{\mathrm{vi}}-I_{\mathrm{vi}}^{\mathrm{low}}\right|,\;\;\;E_{\mathrm{vi}}=\tanh\left(\mathrm{Sobel}\left(I_{\mathrm{vi}}\right)\right),$$

where $M_{\mathrm{vi}}^{k}$ indicates the spatial strength of the *k*-th frequency pattern. $I_{\mathrm{vi}}$, $I_{\mathrm{vi}}^{\mathrm{low}}$, $I_{\mathrm{vi}}^{\mathrm{high}}$, and $E_{\mathrm{vi}}$ provide source intensity, low-frequency, high-frequency, and edge cues from the visible image, while $M_{c}$ provides the consensus structural reference. A frequency pattern is more likely to represent reliable texture or edge-related complementarity when it is consistent with source high-frequency responses, edge structures, and cross-modal consensus. Therefore, the reliability map of the *k*-th frequency pattern is predicted as follows:

(36) $$Q_{\mathrm{vi}}^{k}=\sigma \left(\Psi_{\mathrm{vi}}\left(\left[M_{\mathrm{vi}}^{k},I_{\mathrm{vi}},I_{\mathrm{vi}}^{\mathrm{high}},I_{\mathrm{vi}}^{\mathrm{low}},E_{\mathrm{vi}},M_{c}\right]\right)\right),$$

where $\Psi_{\mathrm{vi}}\left(\cdot \right)$ denotes the visible reliability prediction head, and $Q_{\mathrm{vi}}^{k}\in {\left[0,1\right]}^{1\times H\times W}$ denotes the spatial reliability map of the *k*-th frequency pattern. The verified frequency patterns are then aggregated according to their reliability maps:

(37) $$R_{\mathrm{vi}}^{t}=\sum_{k=1}^{K}Q_{\mathrm{vi}}^{k}⊙R_{\mathrm{vi}}^{k},\;\;\;Q_{\mathrm{vi}}=\frac{1}{K}\sum_{k=1}^{K}Q_{\mathrm{vi}}^{k},$$

where $R_{\mathrm{vi}}^{t}$ denotes the visible reliability-weighted residual after frequency-pattern verification, and $Q_{\mathrm{vi}}$ summarizes the overall visible reliability across frequency patterns. Finally, the visible trustworthy complementary feature is obtained as follows:

(38) $${\tilde{R}}_{\mathrm{vi}}=\Phi_{\mathrm{vi}}\left(\left[R_{\mathrm{vi}}^{t},Q_{\mathrm{vi}}\right]\right),$$

where $\Phi_{\mathrm{vi}}\left(\cdot \right)$ denotes the visible complementary feature mapping function, and ${\tilde{R}}_{\mathrm{vi}}$ denotes the verified visible complementary feature.

To prevent different frequency masks from collapsing into similar responses, we introduce a frequency-pattern diversity constraint:

(39) $$L_{\mathrm{freq}}=\sum_{i<j}\left|\frac{\langle G_{i},G_{j}\rangle }{{∥G_{i}∥}_{2}{∥G_{j}∥}_{2}+\epsilon }\right|,$$

where *i* and *j* denote different frequency-pattern indices, and $\langle G_{i},G_{j}\rangle$ denotes the inner product between two frequency masks on the frequency plane. This constraint discourages excessive overlap among frequency masks and encourages them to cover complementary frequency patterns.

Through TCV, the infrared and visible candidate residuals are transformed into trustworthy complementary features ${\tilde{R}}_{\mathrm{ir}}$ and ${\tilde{R}}_{\mathrm{vi}}$, together with their reliability maps $Q_{\mathrm{ir}}$ and $Q_{\mathrm{vi}}$. These verified residual features and reliability maps provide reliability-aware inputs for subsequent cross-modal conflict estimation.

#### 2.4.3. Cross-Modal Conflict Estimation

**Figure 7:**
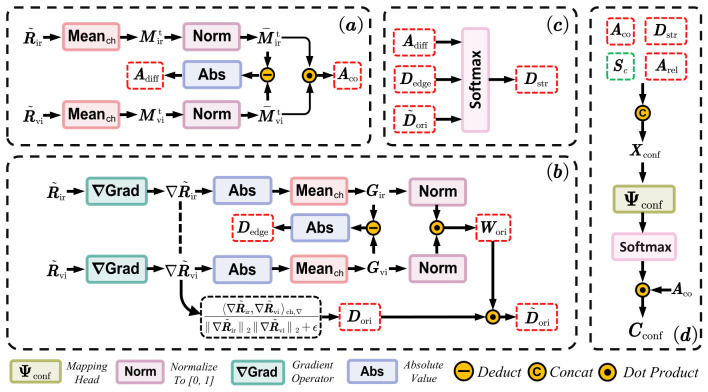
Framework of the proposed Cross-Modal Conflict Estimation: (**a**) co-activation and amplitude-mismatch estimation; (**b**) edge-strength and orientation-mismatch estimation; (**c**) structural-mismatch aggregation; and (**d**) conflict-map prediction. The red dashed boxes highlight the intermediate cue maps used in subsequent aggregation or conflict prediction.

The verified complementary features indicate relatively reliable modality-specific information, but they do not guarantee that infrared and visible complementary responses can be directly superimposed at every spatial position. In particular, two reliable responses may still emphasize inconsistent boundaries, textures, or local structures. Therefore, we propose the CMCE to explicitly estimate the local structural inconsistency between infrared and visible trustworthy complementary features, as shown in Figure 7.

The cue construction is motivated by a local multi-order description of feature structure. The channel-averaged response amplitude is a zero-order statistic that reflects local activation energy; its mismatch indicates that the two trustworthy branches assign substantially different strengths to the same region. Spatial gradients provide first-order structural information. A gradient-magnitude difference measures disagreement in boundary or texture strength, whereas normalized gradient orientation detects directional inconsistency even when the two magnitudes are similar. None of these quantities alone is sufficient to define conflict: amplitude differences can occur in valid modality-specific regions, and orientation comparisons are unreliable in flat areas. CMCE, therefore, combines them only under co-activation and joint-reliability support and further suppresses orientation evidence when valid gradients are absent. This design turns the three cues into complementary measurements of local incompatibility rather than an unconditioned collection of handcrafted differences.

We first compute the channel-averaged magnitude maps of the two trustworthy complementary features and normalize their response scales:

(40) $$M_{\mathrm{ir}}^{t}={\mathrm{Mean}}_{\mathrm{ch}}\left(\right|{\tilde{R}}_{\mathrm{ir}}|),\;\;\;M_{\mathrm{vi}}^{t}={\mathrm{Mean}}_{\mathrm{ch}}\left(\right|{\tilde{R}}_{\mathrm{vi}}\left|\right),$$

(41) $${\bar{M}}_{\mathrm{ir}}^{t}=N_{a}\left(M_{\mathrm{ir}}^{t}\right),\;\;\;{\bar{M}}_{\mathrm{vi}}^{t}=N_{a}\left(M_{\mathrm{vi}}^{t}\right),$$

where $M_{\mathrm{ir}}^{t}$ and $M_{\mathrm{vi}}^{t}$ describe the spatial response intensities of the infrared and visible trustworthy complementary features, respectively. $N_{a}\left(\cdot \right)$ denotes amplitude normalization, which makes the response magnitudes comparable across modalities. Based on the normalized responses, we define the co-activation map and amplitude imbalance map as follows:

(42) $$A_{\mathrm{co}}={\bar{M}}_{\mathrm{ir}}^{t}⊙{\bar{M}}_{\mathrm{vi}}^{t},\;\;\;A_{\mathrm{diff}}=\left|{\bar{M}}_{\mathrm{ir}}^{t}-{\bar{M}}_{\mathrm{vi}}^{t}\right|,$$

where $A_{\mathrm{co}}$ indicates regions where both complementary branches are active, and $A_{\mathrm{diff}}$ measures their normalized response-magnitude imbalance. Co-activation provides a necessary condition for possible conflict, whereas amplitude imbalance offers only a coarse conflict-related cue. Therefore, we further introduce structural evidence from gradient responses.

Next, we compute the gradient magnitude maps of the two trustworthy complementary features and measure their edge-strength mismatch:

(43) $$G_{\mathrm{ir}}={\mathrm{Mean}}_{\mathrm{ch}}(|\nabla {\tilde{R}}_{\mathrm{ir}}|),\;\;\;G_{\mathrm{vi}}={\mathrm{Mean}}_{\mathrm{ch}}(|\nabla {\tilde{R}}_{\mathrm{vi}}\left|\right),$$

(44) $$D_{\mathrm{edge}}=\left|G_{\mathrm{ir}}-G_{\mathrm{vi}}\right|,$$

where ∇ denotes the spatial gradient operator, $G_{\mathrm{ir}}$ and $G_{\mathrm{vi}}$ characterize local boundary or texture strength, and $D_{\mathrm{edge}}$ measures their structural strength discrepancy. However, two responses with similar gradient magnitudes may still point to different local orientations. To capture this case, we further compute the gradient orientation consistency and orientation mismatch:

(45) $$C_{\mathrm{ori}}=\frac{{\langle \nabla {\tilde{R}}_{\mathrm{ir}},\nabla {\tilde{R}}_{\mathrm{vi}}\rangle }_{\mathrm{ch},\nabla }}{{∥\nabla {\tilde{R}}_{\mathrm{ir}}∥}_{2,\mathrm{ch},\nabla }{∥\nabla {\tilde{R}}_{\mathrm{vi}}∥}_{2,\mathrm{ch},\nabla }+\epsilon },\;\;\;D_{\mathrm{ori}}=1-\left|C_{\mathrm{ori}}\right|,$$

where ${\langle \cdot ,\cdot \rangle }_{\mathrm{ch},\nabla }$ and ${∥\cdot ∥}_{2,\mathrm{ch},\nabla }$ are computed over both the channel and gradient-direction dimensions at each spatial position, and ϵ is used for numerical stability. $C_{\mathrm{ori}}$ measures orientation consistency, while $D_{\mathrm{ori}}$ reflects orientation mismatch. The absolute value avoids treating opposite gradient signs with consistent edge orientations as strong conflicts.

Since orientation comparison is meaningful only when both complementary features contain valid gradient responses, we introduce a gradient validity weight:

(46) $$W_{\mathrm{ori}}=N_{a}\left(G_{\mathrm{ir}}\right)⊙N_{a}\left(G_{\mathrm{vi}}\right),\;{\tilde{D}}_{\mathrm{ori}}=W_{\mathrm{ori}}⊙D_{\mathrm{ori}},$$

where $W_{\mathrm{ori}}$ suppresses unreliable orientation comparison in weak-gradient regions, and ${\tilde{D}}_{\mathrm{ori}}$ denotes the validity-weighted orientation mismatch. By combining amplitude imbalance, edge mismatch, and orientation mismatch, CMCE obtains a more comprehensive structural mismatch map:

(47) $$D_{\mathrm{str}}=\omega_{a}N\left(A_{\mathrm{diff}}\right)+\omega_{e}N\left(D_{\mathrm{edge}}\right)+\omega_{o}{\tilde{D}}_{\mathrm{ori}},$$

(48) $$\left[\omega_{a},\omega_{e},\omega_{o}\right]=\mathrm{Softmax}\left(\left[\alpha_{a},\alpha_{e},\alpha_{o}\right]\right),$$

where N(·) normalizes different mismatch cues to comparable scales. $\alpha_{a}$, $\alpha_{e}$, and $\alpha_{o}$ are learnable parameters, and their Softmax-normalized weights $\omega_{a}$, $\omega_{e}$, and $\omega_{o}$ adaptively balance amplitude, edge, and orientation mismatch. In this way, $D_{\mathrm{str}}$ summarizes local structural inconsistency from multiple complementary perspectives. Nevertheless, structural mismatch alone is not sufficient to determine conflict, since conflict should occur mainly where both branches are active and reliable.

To incorporate these conditions, we construct the conflict-estimation input using co-activation, complementary reliability, structural mismatch, and consensus support:

(49) $$A_{\mathrm{rel}}=Q_{\mathrm{ir}}⊙Q_{\mathrm{vi}},$$

(50) $$X_{\mathrm{conf}}=\left[A_{\mathrm{co}},A_{\mathrm{rel}},D_{\mathrm{str}},S_{c}\right],$$

(51) $$C_{\mathrm{conf}}=A_{\mathrm{co}}⊙A_{\mathrm{rel}}⊙\sigma \left(\Psi_{\mathrm{conf}}\left(X_{\mathrm{conf}}\right)\right),$$

where $A_{\mathrm{rel}}$ denotes the joint reliability support of the infrared and visible complementary branches. $X_{\mathrm{conf}}$ provides reliability-aware structural evidence for conflict estimation, $\Psi_{\mathrm{conf}}\left(\cdot \right)$ denotes the conflict-estimation function, and σ(·) denotes the Sigmoid function. The resulting $C_{\mathrm{conf}}\in {\left[0,1\right]}^{1\times H\times W}$ represents the local conflict intensity. Here, $A_{\mathrm{co}}$ restricts conflict estimation to regions where both complementary branches are active, while $A_{\mathrm{rel}}$ suppresses unreliable complementary regions. Meanwhile, $S_{c}$ provides a consensus reference, allowing the network to calibrate whether the observed mismatch should be treated as harmful conflict or acceptable modality-specific variation.

Therefore, CMCE defines conflict as structural inconsistency between trustworthy complementary responses, rather than a simple source-image difference or response-magnitude competition. The obtained conflict map $C_{\mathrm{conf}}$ guides the subsequent routing stage to dynamically arbitrate between cross-modal consensus and modality-specific complementary information.

#### 2.4.4. Conflict-Aware Routing

To adaptively coordinate the consensus feature and modality-specific trustworthy complementary features under local conflict states, we propose the CAR. Rather than directly superimposing the three branches, CAR converts consensus support, complementary reliability, and conflict intensity into routing cues, so that the fusion process can favor stable consensus in conflicting regions while preserving reliable modality-specific information.

We first construct spatial gates for the consensus branch, infrared complementary branch, and visible complementary branch:

(52) $$G_{c}=\sigma \left(\Psi_{c}\left(\left[F_{c},S_{c},C_{\mathrm{conf}}\right]\right)\right),$$

(53) $$G_{\mathrm{ir}}=\sigma \left(\Psi_{\mathrm{ir}}^{g}\left(\left[{\tilde{R}}_{\mathrm{ir}},Q_{\mathrm{ir}},S_{c},C_{\mathrm{conf}}\right]\right)\right),\;G_{\mathrm{vi}}=\sigma \left(\Psi_{\mathrm{vi}}^{g}\left(\left[{\tilde{R}}_{\mathrm{vi}},Q_{\mathrm{vi}},S_{c},C_{\mathrm{conf}}\right]\right)\right),$$

where $\Psi_{c}\left(\cdot \right)$, $\Psi_{\mathrm{ir}}^{g}\left(\cdot \right)$, and $\Psi_{\mathrm{vi}}^{g}\left(\cdot \right)$ are gate prediction functions for the consensus, infrared complementary, and visible complementary branches, respectively. The resulting gates $G_{c}$, $G_{\mathrm{ir}}$, and $G_{\mathrm{vi}}$ provide spatial calibration and are broadcast along the channel dimension. Specifically, the consensus gate uses $S_{c}$ and $C_{\mathrm{conf}}$ to emphasize stable common structures, while the complementary gates additionally exploit $Q_{\mathrm{ir}}$ and $Q_{\mathrm{vi}}$ to control the injection of reliable residual information. Thus, these gates perform branch-wise spatial filtering before inter-branch routing:

(54) $${\bar{F}}_{c}=G_{c}⊙F_{c},\;\;\;{\bar{R}}_{\mathrm{ir}}=G_{\mathrm{ir}}⊙{\tilde{R}}_{\mathrm{ir}},\;\;\;{\bar{R}}_{\mathrm{vi}}=G_{\mathrm{vi}}⊙{\tilde{R}}_{\mathrm{vi}},$$

where ${\bar{F}}_{c}$, ${\bar{R}}_{\mathrm{ir}}$, and ${\bar{R}}_{\mathrm{vi}}$ denote the gated consensus, infrared complementary, and visible complementary features, respectively.

After this intra-branch calibration, CAR further models the competition among the three branches. To this end, the calibrated features and relation cues are concatenated as the routing input:

(55) $$X_{\mathrm{route}}=\left[{\bar{F}}_{c},{\bar{R}}_{\mathrm{ir}},{\bar{R}}_{\mathrm{vi}},S_{c},C_{\mathrm{conf}},Q_{\mathrm{ir}},Q_{\mathrm{vi}},A_{\mathrm{co}}\right],$$

(56) $$\left[\Omega_{c},\Omega_{\mathrm{ir}},\Omega_{\mathrm{vi}}\right]=\Psi_{\mathrm{route}}\left(X_{\mathrm{route}}\right),$$

where $X_{\mathrm{route}}$ collects the calibrated branch features and relation cues for routing prediction, and $A_{\mathrm{co}}$ is the co-activation cue defined in CMCE. $\Psi_{\mathrm{route}}\left(\cdot \right)$ predicts the initial routing logits $\Omega_{c}$, $\Omega_{\mathrm{ir}}$, and $\Omega_{\mathrm{vi}}$ for the consensus, infrared complementary, and visible complementary branches, respectively.

To make the routing behavior explicitly responsive to conflict and reliability, we further introduce non-negative modulation coefficients:

(57) $${\hat{\beta }}_{c}=\mathrm{softplus}\left(\beta_{c}\right),\;\;\;{\hat{\beta }}_{\mathrm{ir}}=\mathrm{softplus}\left(\beta_{\mathrm{ir}}\right),\;\;\;{\hat{\beta }}_{\mathrm{vi}}=\mathrm{softplus}\left(\beta_{\mathrm{vi}}\right),$$

(58) $${\hat{\chi }}_{\mathrm{ir}}=\mathrm{softplus}\left(\chi_{\mathrm{ir}}\right),\;\;\;{\hat{\chi }}_{\mathrm{vi}}=\mathrm{softplus}\left(\chi_{\mathrm{vi}}\right),$$

where $\beta_{c}$, $\beta_{\mathrm{ir}}$, $\beta_{\mathrm{vi}}$, $\chi_{\mathrm{ir}}$, and $\chi_{\mathrm{vi}}$ are learnable modulation parameters, and their hatted versions denote the corresponding non-negative coefficients. The routing logits are then relation-calibrated as follows:

(59) $$\Omega_{c}^{\prime }=\Omega_{c}+{\hat{\beta }}_{c}C_{\mathrm{conf}},$$

(60) $$\Omega_{\mathrm{ir}}^{\prime }=\Omega_{\mathrm{ir}}+{\hat{\beta }}_{\mathrm{ir}}Q_{\mathrm{ir}}-{\hat{\chi }}_{\mathrm{ir}}C_{\mathrm{conf}}\left(1-Q_{\mathrm{ir}}\right),$$

(61) $$\Omega_{\mathrm{vi}}^{\prime }=\Omega_{\mathrm{vi}}+{\hat{\beta }}_{\mathrm{vi}}Q_{\mathrm{vi}}-{\hat{\chi }}_{\mathrm{vi}}C_{\mathrm{conf}}\left(1-Q_{\mathrm{vi}}\right),$$

where $\Omega_{c}^{\prime }$, $\Omega_{\mathrm{ir}}^{\prime }$, and $\Omega_{\mathrm{vi}}^{\prime }$ denote the relation-calibrated routing logits. When local conflict is strong, the consensus branch is encouraged to provide a more stable routing anchor. In contrast, the complementary branches are promoted when their reliability is high, but explicitly suppressed when low reliability coincides with strong conflict. Therefore, the conflict map does not simply discard complementary information; instead, it works together with reliability cues to determine how much each complementary branch should be preserved.

A branch-wise Softmax operation is then applied at each spatial position to obtain the routing weights:

(62) $$\left[W_{c},W_{\mathrm{ir}},W_{\mathrm{vi}}\right]=\mathrm{Softmax}\left(\left[\Omega_{c}^{\prime },\Omega_{\mathrm{ir}}^{\prime },\Omega_{\mathrm{vi}}^{\prime }\right]\right),\;\;\;W_{c}+W_{\mathrm{ir}}+W_{\mathrm{vi}}=1,$$

where $W_{c}$, $W_{\mathrm{ir}}$, and $W_{\mathrm{vi}}\in {\left[0,1\right]}^{1\times H\times W}$ denote the spatial routing weights for the three branches and are broadcast along the channel dimension during feature fusion. The routed C^3^ feature is obtained as follows:

(63) $$F_{C^{3}}=W_{c}⊙{\bar{F}}_{c}+W_{\mathrm{ir}}⊙{\bar{R}}_{\mathrm{ir}}+W_{\mathrm{vi}}⊙{\bar{R}}_{\mathrm{vi}},$$

where $F_{C^{3}}$ denotes the relation-aware feature integrated from consensus, trustworthy complementarity, and conflict-aware routing.

Although $F_{C^{3}}$ captures the calibrated C^3^ relationship, relying only on routed residual information may weaken the basic intensity and structural content of the original encoded features. Therefore, we additionally employ a lightweight base fusion branch:

(64) $$F_{\mathrm{base}}=\Phi_{b}\left(\left[F_{\mathrm{ir}},F_{\mathrm{vi}},S_{c}\right]\right),$$

where $\Phi_{b}\left(\cdot \right)$ denotes the base fusion mapping, and $F_{\mathrm{base}}$ preserves fundamental dual-modal information with the same feature dimension as $F_{C^{3}}$. The final fused feature is defined as:

(65) $$F_{\mathrm{fuse}}=\Phi_{\mathrm{out}}\left(F_{\mathrm{base}}+\lambda_{f}F_{C^{3}}\right),$$

where $\Phi_{\mathrm{out}}\left(\cdot \right)$ denotes the output refinement function, $\lambda_{f}$ is a learnable scaling factor, and $F_{\mathrm{fuse}}$ denotes the final fused feature.

Through CAR, the fusion network relies more on stable consensus in conflict regions, while adaptively preserving infrared salient responses and visible details in reliable complementary regions. This enables ARC^3^Fusion to perform relation-aware feature integration rather than direct response superposition.

### 2.5. Decoder

**Figure 8:**
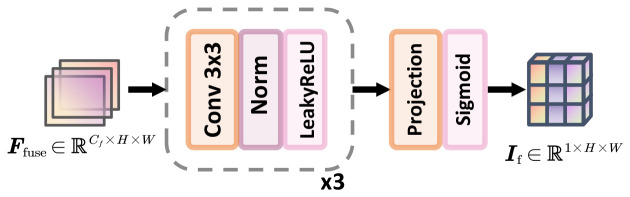
Framework of the proposed Decoder.

The decoder, as shown in Figure 8, maps the fused feature $F_{\mathrm{fuse}}\in R^{C_{f}\times H\times W}$ produced by C^3^ routing back to the single-channel fused image space. Specifically, the decoder consists of three 3×3 convolutional reconstruction layers that preserve the spatial resolution, followed by an output convolution. Given $D_{0}=F_{\mathrm{fuse}}$, the intermediate reconstruction features are defined as follows:

(66) $$D_{l}=\mathrm{LReLU}\left(\mathrm{Norm}\left({\mathrm{Conv}}_{3\times 3}^{l}\left(D_{l-1}\right)\right)\right),\;\;\;l=1,2,3,$$

where Norm(·) denotes the normalization layer, LReLU(·) denotes the LeakyReLU activation function, and all convolutional layers use stride 1 to maintain the spatial resolution.

The final fused image is obtained through an output convolution followed by sigmoid normalization:

(67) $$I_{f}=\sigma \left({\mathrm{Conv}}_{3\times 3}^{\mathrm{out}}\left(D_{3}\right)\right),$$

where σ(·) constrains the output to the range [0,1]. The final network output is a single-channel fused luminance image $I_{f}\in R^{1\times H\times W}$. For RGB visualization, $I_{f}$ is used as the *Y* channel and combined with the Cb and Cr chrominance channels directly inherited from the original visible image. The resulting YCbCr representation is then converted into RGB.

### 2.6. Loss Functions and Constraints

The overall objective consists of a fusion reconstruction loss and an auxiliary constraint loss. The fusion reconstruction loss directly supervises the final fused image to improve its overall visual quality, while the auxiliary constraints act on intermediate relation representations in the fusion core. These auxiliary constraints guide the learnable mappings to follow their expected semantic roles in C^3^ relationship modeling, rather than degenerating into unconstrained feature transformations.

#### 2.6.1. Fusion Loss

Following the max-response intensity and gradient preservation objectives commonly used in infrared–visible image fusion [46], the fusion loss supervises the predicted fused luminance image from three aspects: intensity preservation, gradient detail preservation, and structural similarity. Given the visible luminance image $I_{\mathrm{vi}}$, the infrared image $I_{\mathrm{ir}}$, and the fused luminance image $I_{f}$, the intensity preservation loss is defined as follows:

(68) $$L_{\mathrm{int}}={∥I_{f}-\max\left(I_{\mathrm{ir}},I_{\mathrm{vi}}\right)∥}_{1},$$

where the maximum operation is performed element-wise. This term encourages the fused image to retain the dominant intensity responses from the two source images, thereby preserving salient thermal targets from the infrared image and informative luminance responses from the visible image.

To preserve edges and fine-grained texture details, we employ the Sobel gradient operator [38] to construct the gradient preservation loss:

(69) $$L_{\mathrm{grad}}={∥\left|S(I_{f})\right|-\max\left(\left|S(I_{\mathrm{ir}})\right|,\left|S(I_{\mathrm{vi}})\right|\right)∥}_{1},$$

where S(·) denotes the Sobel gradient operator, and the maximum operation is performed element-wise. By selecting the stronger gradient response at each spatial position as the supervision target, this loss encourages the fused image to preserve complementary edges, textures, and structural details from both modalities.

In addition to intensity and gradient information, local structural consistency is further considered through a modality-adaptive structural similarity loss. Specifically, we construct modality selection masks according to the local standard deviation maps of the infrared and visible images:

(70) $$M_{\mathrm{ir}}=I_{\mathrm{Std}\left(I_{\mathrm{ir}}\right)>\mathrm{Std}\left(I_{\mathrm{vi}}\right)},$$

(71) $$M_{\mathrm{vi}}=1-M_{\mathrm{ir}},$$

where Std(·) denotes the local standard deviation map, and $I_{\left(\cdot \right)}$ denotes the indicator function. The masks $M_{\mathrm{ir}}$ and $M_{\mathrm{vi}}$ identify the spatial regions in which the infrared or visible image exhibits stronger local structural variation and should, therefore, serve as the structural reference.

Based on the modality selection masks, the structural similarity loss is defined using the structural similarity formulation [47]:

(72) $$L_{\mathrm{ssim}}=1-\mathrm{mean}\left(M_{\mathrm{ir}}⊙{\mathrm{SSIM}}_{\mathrm{map}}\left(I_{\mathrm{ir}},I_{f}\right)+M_{\mathrm{vi}}⊙{\mathrm{SSIM}}_{\mathrm{map}}\left(I_{\mathrm{vi}},I_{f}\right)\right),$$

where ${\mathrm{SSIM}}_{\mathrm{map}}\left(\cdot ,\cdot \right)$ denotes the local structural similarity map, ⊙ represents element-wise multiplication, and mean(·) denotes spatial averaging. Rather than uniformly constraining the fused image toward either source modality, this loss adaptively selects the source image with stronger local structural variation as the reference at each spatial position.

The overall fusion loss is formulated as follows:

(73) $$L_{\mathrm{fuse}}=\lambda_{\mathrm{int}}L_{\mathrm{int}}+\lambda_{\mathrm{grad}}L_{\mathrm{grad}}+\lambda_{\mathrm{ssim}}L_{\mathrm{ssim}},$$

where $\lambda_{\mathrm{int}}$, $\lambda_{\mathrm{grad}}$, and $\lambda_{\mathrm{ssim}}$ denote the weighting coefficients of the intensity preservation, gradient preservation, and structural similarity losses, respectively. Through their joint optimization, the fusion loss encourages the generated luminance image to simultaneously preserve prominent intensity responses, complementary gradient details, and locally salient structural information from the infrared and visible modalities.

#### 2.6.2. Auxiliary Loss

For auxiliary constraints, we regularize the intermediate relation evidence in the fusion core to guide the optimization directions of consensus decomposition, candidate residual preservation, consensus-residual decoupling, and frequency-pattern separation.

For CMERD, the regional explanation constraint $L_{\exp}$ suppresses residual responses in high-consensus regions, the residual preservation constraint $L_{\mathrm{res}}$ prevents candidate residuals in low-consensus regions from being over-compressed, and the thresholded consensus-residual decoupling constraint $L_{\mathrm{orth}}$ reduces redundant correlations between consensus features and residual features. For the visible frequency-pattern verification in TCV, the frequency-pattern diversity constraint $L_{\mathrm{freq}}$ reduces the overlap among different frequency masks, encouraging different patterns to undertake more explicit frequency-specific roles.

The auxiliary loss is defined as follows:

(74) $$L_{\mathrm{aux}}=\lambda_{\exp}L_{\exp}+\lambda_{\mathrm{res}}L_{\mathrm{res}}+\lambda_{\mathrm{orth}}L_{\mathrm{orth}}+\lambda_{\mathrm{freq}}L_{\mathrm{freq}},$$

where $\lambda_{\exp}$, $\lambda_{\mathrm{res}}$, $\lambda_{\mathrm{orth}}$, and $\lambda_{\mathrm{freq}}$ denote the weighting coefficients of the auxiliary constraint terms. The final training objective is formulated as follows:

(75) $$L_{\mathrm{total}}=L_{\mathrm{fuse}}+\lambda_{\mathrm{aux}}L_{\mathrm{aux}},$$

where $\lambda_{\mathrm{aux}}$ controls the contribution of the auxiliary constraints to the overall training objective.

With the above objective function, ARC^3^Fusion optimizes the final fused luminance image from the perspectives of intensity preservation, gradient detail preservation, and structural similarity. Meanwhile, the auxiliary constraints explicitly regularize consensus decomposition, candidate residual preservation, consensus–residual decoupling, and frequency-pattern separation. As a result, the learnable mappings in the network can maintain data adaptability while more stably serving the proposed C^3^ relationship modeling objective.

## 3. Experiments and Results

### 3.1. Datasets and Experimental Settings

**Figure 9:**
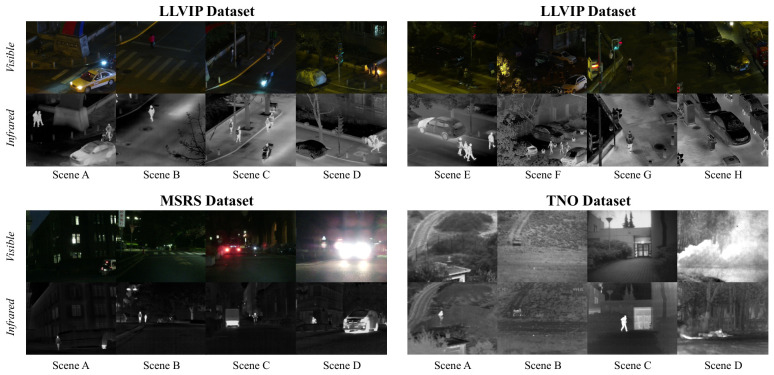
Typical low-illumination scenes of LLVIP, MSRS, and TNO.

**Dataset.** Our experiments are conducted on three classical datasets for infrared and visible image fusion, including LLVIP [48], MSRS [49], and TNO [50]. These datasets cover diverse imaging conditions and scene distributions, providing a suitable basis for evaluating both the in-domain fusion performance and the cross-dataset generalization ability of ARC^3^Fusion. LLVIP and MSRS are adopted for model training and in-domain evaluation. Specifically, we select 3500 paired infrared and visible images from low-illumination scenarios in LLVIP and 700 paired infrared and visible images from low-illumination scenarios in MSRS. For each dataset, all image pairs are randomly shuffled and divided into training and testing subsets at a ratio of 7:3. The training subsets of LLVIP and MSRS are combined to train a unified ARC^3^Fusion model, and their testing subsets are used for in-domain evaluation. TNO is further employed as a cross-dataset generalization benchmark, where 58 representative infrared–visible image pairs are selected from typical fusion scenarios. It should be noted that TNO is not involved in the training process and is only used to evaluate the generalization performance of ARC^3^Fusion under unseen data distributions. Several typical scenes from the three datasets are shown in Figure 9. As observed from the visual examples, the visible images suffer from severe degradation under low-illumination conditions, including low contrast, texture attenuation, and detail loss.

**Experimental Settings.** Our experiments are conducted on a workstation equipped with an NVIDIA A800-80GB-NVLink GPU and an Intel Xeon (R) Platinum 8358 CPU. The proposed model is implemented in Python 3.12 with PyTorch 2.5.1 [51]. During the data preprocessing stage, the input infrared and visible images are resized to 225×225 and normalized before being fed into the network. In addition, all training samples are randomly shuffled to reduce the influence of data ordering and improve training stability. The detailed training hyperparameter settings are summarized in Table 1.

**Baseline Implementations and Evaluation Protocol.** For a fair and reproducible comparison, ARC^3^Fusion and all baseline methods were evaluated on the same hardware platform and software environment using identical infrared–visible test pairs. All competing methods were implemented with their officially released codebases and pretrained weights, without additional retraining, fine-tuning, or parameter adaptation. The same external preprocessing and evaluation protocol was applied to all methods, while the model-specific operations defined in their official inference pipelines were retained. During inference, the original spatial resolution of each input pair was preserved, and the fused output maintained the corresponding input dimensions. All quantitative metrics were calculated using the same implementations, and no additional post-processing was introduced.

### 3.2. Loss Hyperparameter Configuration

Through repeated empirical experiments, we obtained a comparatively favorable configuration of the loss-weighting coefficients, rather than claiming a globally optimal solution. The selected values provide a balanced trade-off among intensity preservation, gradient-detail enhancement, structural consistency, and intermediate representation regularization. The detailed configuration is reported in Table 2. All subsequent comparative experiments and quantitative metric evaluations are conducted using the model trained with this loss configuration.

The fusion-loss terms are assigned relatively larger weights because they directly supervise the final fused luminance image and determine the preservation of source intensity, gradient details, and local structures. In comparison, the contribution of the auxiliary objective is controlled at a relatively limited level with respect to the main fusion objective, allowing the intermediate semantic constraints to regularize the C^3^ relation representations without excessively interfering with the optimization of the final fused result. Equal weights are assigned to the explainability and residual-preservation losses to balance the suppression of redundant residual responses in high-consensus regions and the retention of complementary information in low-consensus regions. The orthogonality loss is assigned a small weight because it mainly serves as a soft decorrelation regularizer, rather than enforcing complete independence between the consensus and residual representations. Similarly, the frequency-separation loss is moderately weighted to encourage distinguishable frequency responses while preventing the frequency constraint from dominating the overall optimization.

### 3.3. Comparative Studies

#### 3.3.1. Qualitative Analysis

In the qualitative comparison, ARC^3^Fusion is evaluated against representative infrared–visible image fusion methods, including CDDFuse [20], CrossFuse [21], MUFusion [22], PIAFusion [23], SwinFusion [24], DAFusion [52], and RPFNet [53]. These methods cover feature decomposition, cross-modal interaction, illumination-aware fusion, Transformer-based global modeling, domain-adaptation-guided fusion, and residual-prior frequency-aware modeling. Spatially aligned enlarged regions are provided in the qualitative figures to facilitate the examination of target contours, object boundaries, text patterns, and fine textures.

**Figure 10:**
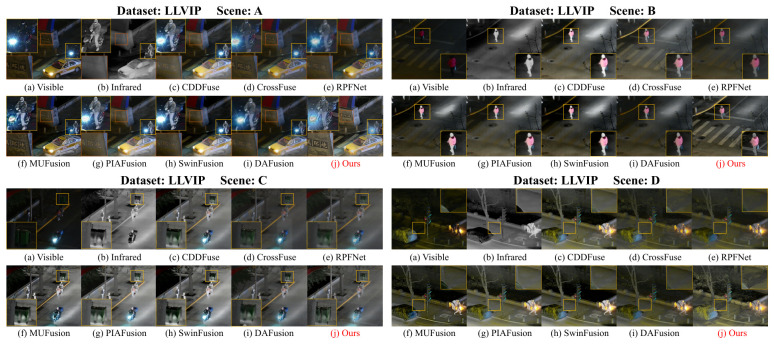
Qualitative comparison of fusion results across Scenes A–D on the LLVIP dataset. Yellow boxes indicate regions of interest, and the enlarged boxes show the corresponding local details.

As shown in Figure 10, we first select representative low-illumination scenes from the LLVIP dataset to analyze the fusion behavior under challenging nighttime conditions. In Scene A, the enlarged regions focus on the textual symbols in the background and the local contour around the motorcycle light, where strong illumination interference can easily introduce halo artifacts or structural distortion. Compared with other methods, ARC^3^Fusion preserves a clearer and more complete contour around the high-intensity light region, while avoiding obvious halo effects. Meanwhile, the textual symbols in the background are also better maintained. This indicates that the proposed method does not simply enhance high-response regions; instead, the reliability verification and conflict-aware routing mechanisms help suppress unreliable local activations while preserving useful visible-domain details.

In Scene B, the pedestrian should be highlighted as an infrared-dominant salient target, whereas the visible textures on the target surface and the surrounding lane markings should also be retained. ARC^3^Fusion achieves a more balanced visual result by simultaneously preserving thermal saliency and visible structural cues. This behavior is consistent with the design of CMERD and TCV: candidate residuals are not directly injected into the fused feature, but are first decomposed and verified before being used as trustworthy complementary information. As a result, the fused image avoids being overly biased toward either infrared saliency or visible texture.

In Scene C, the distant trash bin is more clearly separated from the road background in the result of ARC^3^Fusion, with a more distinguishable boundary and richer local structure. By contrast, several competing methods tend to merge the object with the surrounding road region, resulting in weakened object-background contrast and ambiguous structural hierarchy. This visual difference reflects the role of the consensus feature as a structural anchor. By modeling cross-modal consensus and retaining reliable modality-specific residuals, ARC^3^Fusion can better preserve weak but meaningful object structures in low-contrast regions.

In Scene D, non-thermal background regions are expected to inherit more visible-domain texture information rather than being dominated by homogeneous infrared responses. ARC^3^Fusion better preserves the shadow structures of the trees and maintains more natural background textures. This suggests that the proposed routing mechanism adaptively adjusts modality contributions according to local reliability and conflict states. Overall, the LLVIP visual results show that ARC^3^Fusion does not merely pursue stronger target saliency or sharper edges. Instead, it produces more reliable texture preservation, clearer object-background separation, and more coordinated integration of infrared saliency, visible details, and cross-modal consensus structures, which is aligned with the proposed reliability-calibrated C^3^ fusion design.

**Figure 11:**
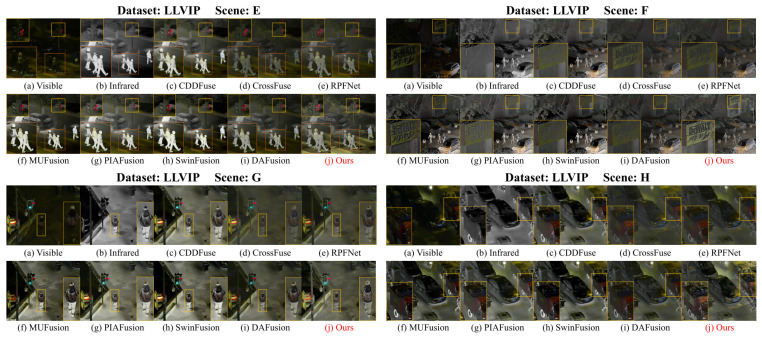
Qualitative comparison of fusion results across Scenes E–H on the LLVIP dataset. Yellow boxes indicate regions of interest, and the enlarged boxes show the corresponding local details.

Beyond the above examples, we further analyze Scenes E–H from LLVIP to evaluate the behavior of ARC^3^Fusion across more diverse nighttime scenarios, as shown in Figure 11. In Scene E, the traffic light, surrounding foliage, and sidewalk bricks belong to different structural layers. ARC^3^Fusion preserves clearer separability among these layers, whereas other methods tend to over-smooth local boundaries and make adjacent structures visually entangled. This indicates that the proposed method maintains local structural stratification rather than simply amplifying visually prominent responses.

In Scene F, the advertising board is used to evaluate fine-grained textual fidelity. ARC^3^Fusion produces more legible characters with fewer blurred or distorted strokes, suggesting that visible-domain details can be reliably preserved even under low illumination. This behavior is consistent with the role of trustworthy complementarity verification, which is designed to distinguish useful texture responses from pseudo-texture artifacts.

In Scene G, both the pedestrian and the ground surface are important for evaluating local detail discrimination. The fused result of ARC^3^Fusion better preserves clothing textures on the pedestrian while maintaining clearer ground details. This demonstrates that the proposed method can adaptively allocate modality contributions between target-related responses and background structures, rather than uniformly favoring one modality across the whole image. In Scene H, under severe low-illumination conditions, ARC^3^Fusion makes the driver, vehicle symbols, and vehicle components more distinguishable than the competing methods. These observations are consistent with the improved visual robustness of ARC^3^Fusion in complex nighttime scenes.

**Figure 12:**
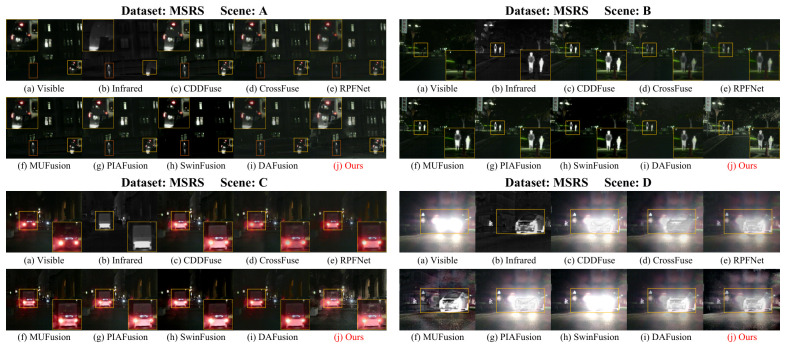
Qualitative comparison of fusion results across Scenes A–D on the MSRS dataset. Yellow boxes indicate regions of interest, and the enlarged boxes show the corresponding local details.

**Figure 13:**
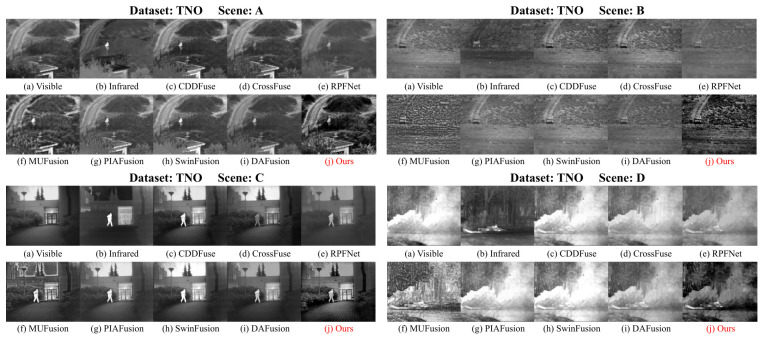
Qualitative comparison of fusion results across Scenes A–D on the TNO dataset.

After the detailed analysis on LLVIP, we further select typical visual examples from MSRS and TNO to evaluate whether ARC^3^Fusion can maintain stable fusion behavior under different scene distributions, as shown in Figure 12 and Figure 13. The results are generally consistent with the observations on LLVIP. On the MSRS scenes, ARC^3^Fusion preserves visible structural details while maintaining salient infrared targets, leading to fused images with clearer local boundaries and more natural texture transitions. This suggests that the proposed reliability-aware fusion strategy remains effective beyond extremely low-light scenarios.

On the TNO scenes, although this dataset is not involved in the training process, ARC^3^Fusion still produces visually stable fused images with clear object-background separation and natural structural transitions. This indicates favorable cross-dataset generalization ability. In particular, for challenging scenes with strong illumination or smoke interference, such as Scene D of MSRS and Scene D of TNO, the proposed method maintains robust fusion performance and generates visually reliable results. This robustness benefits from the fact that ARC^3^Fusion does not directly treat all cross-modal differences as useful information. Instead, candidate residuals are verified, structural conflicts are estimated, and the final fusion is performed through conflict-aware routing.

It is worth noting that ARC^3^Fusion does not always produce the strongest infrared target saliency among all methods. However, the thermal targets remain sufficiently highlighted for visual perception, and the fused images preserve more visible-domain textures and background semantics. Excessive infrared dominance may lead to homogeneous intensity blocks and reduced scene fidelity, especially in non-thermal background regions. Therefore, the visual results suggest that ARC^3^Fusion achieves a more favorable balance among infrared saliency, visible detail preservation, and cross-modal structural consistency. This visual behavior further supports the core motivation of the proposed reliability-calibrated C^3^ fusion framework: reliable fusion should not be direct response superposition, but relation-aware integration guided by consensus, trustworthy complementarity, and conflict estimation.

#### 3.3.2. Quantitative Analysis

For quantitative evaluation, we calculate seven commonly used objective metrics on the testing sets of LLVIP, MSRS, and TNO, including entropy (EN) [54], spatial frequency (SF) [55], average gradient (AG) [56], standard deviation (SD) [57], visual information fidelity (VIF) [58], correlation coefficient (CC) [57], and sum of correlations of differences (SCD) [59]. EN measures the amount of information contained in the fused image. SF reflects spatial activity and texture variation, while AG evaluates gradient-level sharpness and detail preservation capability. SD characterizes the contrast and intensity dispersion of the fused image. VIF measures the amount of visual information preserved from the source images from the perspective of human visual perception. CC evaluates the global correlation between the fused image and the source images, and SCD measures the complementary information transferred from the source images to the fused result. For all these metrics, a higher value generally indicates better fusion performance. The detailed quantitative results are reported in Table 3, Table 4 and Table 5. For each metric, the best and second-best results are highlighted in **RED** and **BLUE**, respectively.

Each table entry is the arithmetic mean of the corresponding image-wise metric over all paired samples in the testing set.

**Figure 14:**
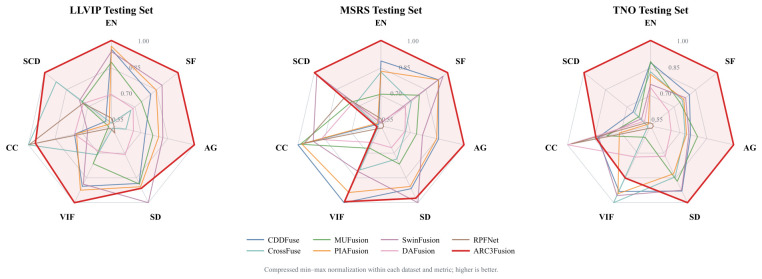
Radar-chart comparison of fusion performance on the LLVIP, MSRS, and TNO testing sets.

Overall, ARC^3^Fusion achieves competitive performance across the three testing sets, with the strongest advantages concentrated in information content, spatial activity, gradient detail, and complementary-information transfer. On LLVIP, it ranks first in EN, SF, AG, VIF, and SCD and second in SD. On MSRS, it ranks first in EN, SF, AG, and SCD and second in SD and VIF. On the unseen TNO dataset, it ranks first in EN, SF, AG, SD, and SCD. These results show that the proposed model maintains favorable performance in both in-domain and cross-dataset evaluation, particularly for detail-oriented and complementarity-oriented metrics. The quantitative trends are consistent with the intended design of ARC^3^Fusion: reliable modality-specific information is retained while local conflicts are coordinated, enabling the fused images to preserve visible texture details and infrared target saliency without an exclusive bias toward either modality. To facilitate a more intuitive comparison, the quantitative results are also presented as radar charts in Figure 14.

A mechanism-level comparison further clarifies these trends. Compared with the decomposition-based CDDFuse [20], ARC^3^Fusion improves SF, AG, VIF, and SCD on LLVIP by 4.078, 1.918, 0.123, and 0.245, respectively. On TNO, the corresponding gains over CDDFuse are 5.738 in SF, 3.235 in AG, 3.413 in SD, and 0.870 in SCD. These improvements suggest that decomposition alone is insufficient to determine whether modality-specific differences should be retained; residual verification and conflict-aware coordination help transform the decomposed differences into more effective detail preservation and complementary-information transfer. Compared with the Transformer-based SwinFusion [24], ARC^3^Fusion obtains higher values on six of the seven LLVIP metrics, whereas SwinFusion retains the highest SD. On MSRS, ARC^3^Fusion exceeds the illumination-aware PIAFusion [23] on six metrics but remains lower in CC. Relative to the recent DAFusion [52] and RPFNet [53] baselines, ARC^3^Fusion achieves higher values than DAFusion on all seven LLVIP metrics and higher values than RPFNet on six metrics. It also outperforms both methods on six of the seven metrics on MSRS and TNO. The higher CC values achieved by several baselines indicate stronger global correlation with the source images, whereas the more consistent advantages of ARC^3^Fusion in EN, SF, AG, and SCD reflect its emphasis on information richness, gradient-detail preservation, and complementary-information transfer.

ARC^3^Fusion does not achieve the best value on every metric, particularly CC on MSRS and VIF on TNO. This mainly reflects the different emphases of the evaluation criteria: CC favors global correlation with the source images, whereas VIF emphasizes perceptual information fidelity. Methods that inherit one source more directly or preserve stronger global similarity may, therefore, obtain higher values on these criteria. ARC^3^Fusion instead coordinates verified modality-specific information to balance visible texture details and infrared thermal saliency. Its consistent advantages in EN, SF, AG, and SCD across the three datasets are aligned with this design objective and indicate effective preservation of informative content, spatial details, and cross-modal complementarity.

### 3.4. Ablation Study

To assess the contribution of the proposed components and examine whether their effects remain observable across different scene distributions, we conduct module-wise ablation experiments on the LLVIP, MSRS, and TNO testing sets. As reported in Table 6, Table 7 and Table 8, seven variants are constructed by individually removing the source-cue-guided encoder block, modality-specific residual adaptation, consensus-residual decomposition, trustworthy complementarity verification, conflict estimation, conflict-aware routing, or auxiliary relationship constraints. All variants are retrained under the same optimization settings as the full model and evaluated using identical preprocessing, inference resolutions, and metric implementations.

Across the three datasets, the complete model consistently achieves the highest SF, AG, and SCD values, and it also obtains the highest VIF on LLVIP and MSRS. These metrics directly reflect spatial activity, gradient-detail preservation, complementary-information transfer, and perceptual information retention, which are closely aligned with the objective of balancing visible textures and infrared thermal saliency. Several removal variants produce slightly higher values on individual EN, SD, CC, or TNO VIF measurements. Such isolated changes reflect the different emphases of the metrics: entropy and standard deviation favor information dispersion and contrast, whereas CC and VIF emphasize source correlation and perceptual fidelity. Relaxing a verification or routing constraint can increase one of these statistics while reducing detail-oriented or complementarity-oriented measures. Therefore, the ablation results are interpreted jointly across metrics rather than by requiring the complete model to maximize every individual criterion.

CMERD and CAR show the most pronounced and cross-dataset influence on the core detail-oriented metrics. Removing CMERD decreases SF and AG by 2.033 and 0.564 on LLVIP, 1.185 and 0.481 on MSRS, and 1.878 and 1.009 on TNO, respectively. SCD also decreases by 0.118, 0.156, and 0.204. Since CMERD defines the consensus-conditioned residuals $R_{m}=F_{m}-{\hat{F}}_{m\leftarrow n}$ that feed the subsequent relation modeling stages, weakening this upstream decomposition reduces both the recoverable local detail and the transfer of modality-specific information. Removing CAR reduces SF and AG by 1.306 and 0.439 on LLVIP, 0.778 and 0.343 on MSRS, and 1.917 and 0.894 on TNO, while SCD decreases by 0.072, 0.087, and 0.142. This confirms that estimating reliable relation cues alone is insufficient unless they are converted into spatially adaptive fusion decisions.

TCV and CMCE provide complementary calibration effects. Without TCV, SF, AG, and SCD decrease by 0.392, 0.163, and 0.032 on LLVIP; 0.303, 0.083, and 0.052 on MSRS and 1.301, 0.484, and 0.147 on TNO. Although the removal variant obtains higher EN or selected correlation-oriented values in some cases, its consistent loss in spatial activity, gradient detail, and complementary-information transfer indicates that modality-specific residuals benefit from reliability verification before preservation. Removing CMCE similarly lowers SF, AG, and SCD on all three datasets, with reductions of 0.739, 0.096, and 0.007 on LLVIP; 0.614, 0.187, and 0.070 on MSRS and 0.784, 0.305, and 0.065 on TNO. These results support the use of explicit structural-conflict evidence to prevent locally incompatible but individually reliable responses from being combined without coordination.

The source-cue-guided encoder and MSRA also improve the overall balance by providing source-aware evidence and maintaining modality sensitivity before decomposition. Their removal reduces SF, AG, VIF, and SCD in most settings, with the reductions becoming more evident on detail-rich or cross-dataset scenes. The auxiliary constraints have the smallest influence on SF, AG, and SCD, and several global statistics change only marginally when they are removed. This behavior is consistent with their intended role as soft intermediate regularizers rather than dominant reconstruction objectives. Overall, the cross-dataset trends support the sequential design of ARC^3^Fusion: source-aware features establish comparable inputs, CMERD constructs candidate relations, TCV verifies their reliability, CMCE identifies local incompatibility, and CAR converts these estimates into adaptive fusion weights.

### 3.5. Visualization and Interpretation of Learned Relation Maps

To further examine the semantic validity of the learned relation representations, we select a representative scene and visualize the consensus map together with the amplitude, edge-strength, and orientation mismatch cues used in CMCE. All maps are processed using the same min–max normalization strategy and displayed as heatmaps, where colors ranging from blue to red indicate progressively increasing normalized response magnitudes. This visualization facilitates the spatial interpretation of each relation cue and enables a qualitative assessment of whether its response distribution is consistent with the intended role in consensus and cross-modal conflict modeling.

**Figure 15:**
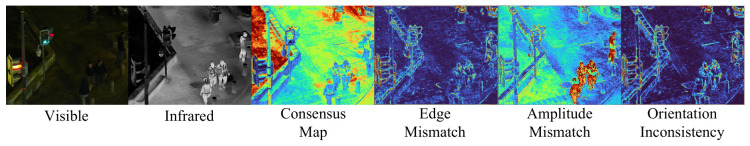
Visualization of consensus and mismatch maps.

As shown in Figure 15, the learned relation maps exhibit spatial distributions consistent with their intended roles. The consensus map produces strong and spatially continuous responses in regions with stable cross-modal correspondence. In particular, the grass area on the left shows the highest consensus support because its broad spatial structure and boundary layout are represented consistently in both modalities, despite the absence of strong target saliency. In contrast, the thermally salient pedestrians receive relatively weak consensus responses, indicating that their dominant infrared activations are treated as modality-specific information rather than shared structures. The edge mismatch map mainly highlights pedestrian contours, road boundaries, traffic-light structures, and other fine object edges, where the trustworthy complementary features exhibit different gradient strengths. The amplitude mismatch map shows stronger region-level responses over thermal targets and areas with substantial cross-modal differences in feature intensity, distinguishing response-strength imbalance from boundary discrepancy. Although the orientation mismatch map presents a distribution similar to the edge mismatch map, its responses are noticeably sparser and concentrate on locations where both modalities contain valid edges but exhibit competing gradient directions. It, therefore, emphasizes rapidly changing structural contours while suppressing flat regions with weak directional evidence. Overall, the distinct yet complementary response patterns support the semantic validity of the consensus representation and the multi-cue conflict modeling in CMCE.

### 3.6. Pixel-Intensity Analysis

**Figure 16:**
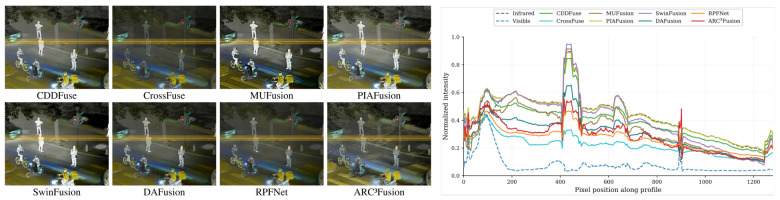
Pixel-intensity analysis on a representative LLVIP scene.

To provide direct pixel-level evidence for the effectiveness of the proposed method, we conduct a representative strip-averaged pixel-intensity analysis on an LLVIP scene. As shown in Figure 16, a horizontal strip with a height of 60 pixels is selected across multiple regions containing visible-light structures, infrared-salient responses, ground textures, and prominent structural boundaries. The same spatial strip is applied to the registered source images and representative fusion results to ensure spatially consistent comparison. For consistent pixel-wise comparison, all source and fusion images are represented in a common single-channel intensity space and normalized to [0, 1] according to their original data ranges, without image-wise min–max stretching or additional post-processing. Following variable-width line-profile analysis, in which neighboring pixels are averaged to obtain a more stable representation of local intensity variation [60], the pixel values at the same horizontal position are averaged across the strip height to reduce the influence of isolated fluctuations and characterize regional intensity changes.

For each source image or fusion method *m*, the strip-averaged pixel-intensity profile is calculated as

(76) $$p_{m}\left(x\right)=\frac{1}{B}\sum_{j=0}^{B-1}{\hat{I}}_{m}\left(x,\;y_{s}+j\right),\;\;\;x=0,\ldots ,W-1,\;\;\;B=60,$$

where $p_{m}\left(x\right)$ denotes the averaged normalized intensity of method *m* at horizontal position *x*, ${\hat{I}}_{m}\in {\left[0,1\right]}^{H\times W}$ denotes the normalized single-channel intensity representation, $y_{s}$ denotes the upper boundary of the selected strip, *B* denotes the strip height, and *W* denotes the image width. Using the same strip for all methods preserves spatial correspondence, while averaging the responses along the vertical direction reduces the sensitivity to isolated pixels and enables a more stable comparison of target–background contrast, local texture variation, and boundary transitions.

As shown in Figure 16, ARC^3^Fusion exhibits different response behaviors according to the local information characteristics. In the visible-dominant region at approximately 0–150 pixels, its profile follows the principal rise and decay of the visible curve, indicating that the local luminance variation and visible structural response are effectively transferred. In the infrared-salient region at approximately 413–475 pixels, the infrared source produces a broad high-response interval. ARC^3^Fusion preserves a continuous response with sufficient target–background contrast, while avoiding the stronger tendency of CDDFuse [20] and SwinFusion [24] to closely reproduce or exceed the infrared intensity distribution. This behavior suggests that the proposed routing process does not simply copy the locally strongest source response, but moderates infrared saliency according to the available complementary and structural evidence.

The interval from approximately 500 to 900 pixels corresponds mainly to the tree pit and its surrounding ground surface. The visible profile contains continuous fine-scale fluctuations associated with the tree-pit boundary and ground textures. CDDFuse and SwinFusion predominantly follow the smoother infrared trend, whereas the RPFNet [53] profile is comparatively flat, and therefore, reflects weaker local variation. By contrast, ARC^3^Fusion retains more visible-related local extrema and variation directions while maintaining a stable overall intensity level. At the salient structural transition around 890–905 pixels, its profile produces a concentrated response at the corresponding source-image boundary without a spatially separated double transition. These observations are consistent with the intended roles of trustworthy complementarity verification and conflict-aware routing: reliable visible details are retained in texture-dominant regions, infrared responses remain sufficiently salient in thermal regions, and locally competing structures are coordinated before final integration.

Overall, the profile of ARC^3^Fusion does not uniformly replicate either source modality. Instead, it changes with the local information content and achieves a coordinated response among visible texture, infrared saliency, and shared structural boundaries. Although a representative profile alone does not constitute dataset-wide statistical evidence, it provides direct pixel-level support that complements the relation-map visualization and module-wise ablation results, and further demonstrates that the proposed reliability verification, conflict estimation, and adaptive routing operate consistently with their intended design.

### 3.7. Computational Complexity and Inference Cost

To further quantify the computational demand of ARC^3^Fusion under practical full-resolution inference, we evaluate its floating-point operations (FLOPs), inference latency, and peak GPU memory consumption on LLVIP, MSRS, and TNO. The evaluation is conducted under the same hardware and software environment described in Section 3.1. We conduct computational complexity evaluations on the LLVIP, MSRS, and TNO testing sets, while retaining the original spatial resolution of each infrared–visible image pair during inference. Specifically, the input resolutions are 1280×1024 for LLVIP, 640×480 for MSRS, and 360×270 for TNO, and the fused outputs preserve the same spatial dimensions as their corresponding inputs. To improve the reliability of the runtime measurements and reduce the influence of initialization overhead and random fluctuations, the first three image pairs of each testing set are used only for GPU warm-up and are excluded from the statistical results. Timing starts from the fourth image pair, and the FLOPs, inference latency, and peak GPU memory consumption are averaged over all remaining test samples to obtain the final efficiency measurements.

ARC^3^Fusion contains 1.172 million trainable parameters.

As shown in Table 9, both the computational cost and GPU memory consumption increase with the spatial resolution of the input image pairs. This resolution-dependent behavior is consistent with the overall architecture of ARC^3^Fusion, which performs feature extraction and consensus–complementarity–conflict relationship modeling while preserving the spatial resolution of the intermediate representations. The reported results provide a practical reference for deploying ARC^3^Fusion in offline processing and server–side infrared–visible image fusion scenarios. Meanwhile, the observed computational overhead also motivates future research on lightweight architectural design and efficient inference for latency-sensitive applications.

## 4. Conclusions

This study investigates the hypothesis that cross-modal differences should not be preserved unconditionally in infrared and visible image fusion. ARC^3^Fusion first separates jointly supported structure from modality-specific candidate residuals, verifies whether those residuals are reliable, and then coordinates verified information when local structures conflict. Experiments on LLVIP, MSRS, and TNO show two main findings: reliability verification improves the preservation of useful saliency and texture while suppressing unstable responses, and conflict-aware coordination produces a more balanced integration of infrared targets and visible structures. The comparative, ablation, relation-map, and pixel-intensity results collectively support this relationship-based view of fusion.

The current method also has limitations. First, ARC^3^Fusion contains multiple relation-modeling and reliability-verification components; how to further simplify the architecture and reduce computational and memory costs while preserving fusion performance remains an important research direction. Second, the present evaluation focuses on fusion quality and has not yet directly verified the benefits of the fused images in downstream high-level vision tasks. Future work will, therefore, investigate lightweight and low-latency implementations and jointly evaluate or optimize the fusion model with downstream detection, segmentation, and remote-sensing perception tasks.

## Figures and Tables

**Table 1:** Training hyperparameter settings.

Parameters	Setup
Optimizer	Adam
Epoch	40
Batch size	4
Warmup epoch	8
Gradient accumulation steps	8
Learning rate	$8\times 10^{-5}$
Weight decay	$1\times 10^{-5}$

**Table 2:** Hyperparameter configuration of the proposed loss function.

Category	Parameter	Value
Fusion loss	$\lambda_{\mathrm{int}}$	18.0
	$\lambda_{\mathrm{grad}}$	25.0
	$\lambda_{\mathrm{ssim}}$	10.0
Auxiliary loss	$\lambda_{\mathrm{aux}}$	1.2
	$\lambda_{\exp}$	1.0
	$\lambda_{\mathrm{res}}$	1.0
	$\lambda_{\mathrm{orth}}$	0.1
	$\lambda_{\mathrm{freq}}$	0.2

**Table 3:** Quantitative comparison of fusion performance on the LLVIP testing set.

Dataset: LLVIP Testing Set							
Method	EN↑	SF↑	AG↑	SD↑	VIF↑	CC↑	SCD↑
CDDFuse	7.047	10.389	2.413	40.795	1.013	0.655	0.984
CrossFuse	6.109	7.390	1.661	21.110	0.776	**0.701**	**1.183**
MUFusion	6.882	8.991	2.711	41.027	0.843	0.621	1.080
PIAFusion	**7.085**	11.200	2.940	41.962	**1.042**	0.653	0.970
SwinFusion	7.018	**12.082**	**3.100**	**47.670**	0.997	0.642	1.089
DAFusion	6.473	7.728	1.952	30.489	0.752	0.655	1.074
RPFNet	6.172	4.785	1.195	22.832	0.577	**0.700**	1.075
**ARC^3^Fusion**	**7.158**	**14.467**	**4.331**	**42.515**	**1.136**	0.694	**1.229**

*Note:* ↑ indicates that a higher value is better. The best and second-best values are shown in bold red and bold blue, respectively. The bold method name identifies the proposed ARC^3^Fusion model.

**Table 4:** Quantitative comparison of fusion performance on the MSRS testing set.

Dataset: MSRS Testing Set							
Method	EN↑	SF↑	AG↑	SD↑	VIF↑	CC↑	SCD↑
CDDFuse	**6.043**	9.089	**2.832**	33.988	**1.131**	**0.693**	0.920
CrossFuse	5.819	6.816	1.839	26.664	0.857	0.630	1.165
MUFusion	5.366	7.482	2.170	27.942	0.676	**0.691**	1.200
PIAFusion	5.834	9.082	2.775	33.454	1.046	0.690	0.932
SwinFusion	4.777	**9.468**	2.348	**37.443**	0.873	0.681	**1.548**
DAFusion	4.797	6.339	1.822	23.894	0.627	0.675	1.186
RPFNet	4.866	4.468	1.181	19.116	0.508	0.688	1.263
**ARC^3^Fusion**	**6.461**	**9.838**	**3.596**	**36.354**	**1.123**	0.631	**1.573**

*Note:* ↑ indicates that a higher value is better. The best and second-best values are shown in bold red and bold blue, respectively. The bold method name identifies the proposed ARC^3^Fusion model.

**Table 5:** Quantitative comparison of fusion performance on the TNO testing set.

Dataset: TNO Testing Set							
Method	EN↑	SF↑	AG↑	SD↑	VIF↑	CC↑	SCD↑
CDDFuse	7.088	**12.852**	4.833	**43.048**	0.745	0.582	**1.242**
CrossFuse	6.962	11.428	4.425	38.904	**0.794**	0.506	1.218
MUFusion	**7.102**	10.867	**5.364**	40.185	0.509	0.568	1.139
PIAFusion	6.925	11.689	4.539	37.974	0.755	0.549	0.973
SwinFusion	6.788	12.063	4.719	42.820	**0.763**	0.580	1.093
DAFusion	6.745	8.548	3.376	32.844	0.595	**0.627**	1.040
RPFNet	6.256	5.239	2.041	24.647	0.470	**0.621**	1.005
**ARC^3^Fusion**	**7.391**	**18.590**	**8.068**	**46.461**	0.687	0.584	**2.112**

*Note:* ↑ indicates that a higher value is better. The best and second-best values are shown in bold red and bold blue, respectively. The bold method name identifies the proposed ARC^3^Fusion model.

**Table 6:** Ablation study of different components on the LLVIP testing set.

Dataset: LLVIP Testing Set							
Configurations	EN↑	SF↑	AG↑	SD↑	VIF↑	CC↑	SCD↑
w/o SCS-CGLB	6.986	13.344	4.069	42.261	1.079	0.683	1.190
w/o MSRA	7.026	13.915	4.146	41.668	1.103	0.682	1.220
w/o CMERD	6.889	12.434	3.767	40.378	1.053	0.663	1.111
w/o TCV	**7.185**	14.075	4.168	42.457	**1.131**	0.687	1.197
w/o CMCE	7.154	13.728	4.235	**42.795**	1.123	**0.696**	1.222
w/o CAR	6.947	13.161	3.892	41.123	1.080	0.666	1.157
w/o Aux.	**7.166**	**14.363**	**4.282**	**42.600**	1.130	**0.695**	**1.224**
**ARC^3^Fusion**	7.158	**14.467**	**4.331**	42.515	**1.136**	0.694	**1.229**

*Note:* ↑ indicates that a higher value is better. The best and second-best values are shown in bold red and bold blue, respectively. The bold method name identifies the proposed ARC^3^Fusion model.

**Table 7:** Ablation study of different components on the MSRS testing set.

Dataset: MSRS Testing Set							
Configurations	EN↑	SF↑	AG↑	SD↑	VIF↑	CC↑	SCD↑
w/o SCS-CGLB	6.393	9.457	3.500	35.700	1.095	**0.634**	1.545
w/o MSRA	6.297	9.585	3.406	35.243	1.100	0.614	1.531
w/o CMERD	6.077	8.653	3.115	34.238	1.046	0.613	1.417
w/o TCV	**6.513**	9.535	3.513	**37.175**	1.114	0.632	1.521
w/o CMCE	6.315	9.224	3.409	35.802	1.085	**0.638**	1.503
w/o CAR	6.306	9.060	3.253	35.674	1.058	0.609	1.486
w/o Aux.	**6.475**	**9.835**	**3.595**	**36.583**	**1.119**	0.629	**1.568**
**ARC^3^Fusion**	6.461	**9.838**	**3.596**	36.354	**1.123**	0.631	**1.573**

*Note:* ↑ indicates that a higher value is better. The best and second-best values are shown in bold red and bold blue, respectively. The bold method name identifies the proposed ARC^3^Fusion model.

**Table 8:** Ablation study of different components on the TNO testing set.

Dataset: TNO Testing Set							
Configurations	EN↑	SF↑	AG↑	SD↑	VIF↑	CC↑	SCD↑
w/o SCS-CGLB	**7.431**	18.120	7.920	46.200	**0.694**	**0.590**	2.085
w/o MSRA	7.338	17.891	7.627	45.501	0.679	0.585	2.011
w/o CMERD	7.135	16.712	7.059	43.373	0.654	0.571	1.908
w/o TCV	7.317	17.289	7.584	45.996	**0.695**	**0.589**	1.965
w/o CMCE	7.378	17.806	7.763	44.912	0.682	0.571	2.047
w/o CAR	7.088	16.673	7.174	44.279	0.662	0.577	1.970
w/o Aux.	**7.410**	**18.584**	**8.066**	**46.550**	0.690	0.585	**2.108**
**ARC^3^Fusion**	7.391	**18.590**	**8.068**	**46.461**	0.687	0.584	**2.112**

*Note:* ↑ indicates that a higher value is better. The best and second-best values are shown in bold red and bold blue, respectively. The bold method name identifies the proposed ARC^3^Fusion model.

**Table 9:** Computational complexity and inference cost of ARC^3^Fusion on different testing sets.

Dataset	Input/Output Resolution	FLOPs (GFLOPs)	Inference Time (ms/pair)	Peak GPU Memory (MiB)
LLVIP	1280×1024	4426.962	1511.480	13,610.533
MSRS	640×480	1037.569	334.599	3194.111
TNO	360×270	685.245	210.676	2115.744

## Data Availability

All training and testing datasets utilized in the experiments of our study are open-source [48,49,50].

## Appendix A. Component Roles and Design Considerations

Table A1 provides a compact summary of the function, necessity, and main limitation of each component in the complete ARC^3^Fusion inference pipeline.

**Table A1:** Compact summary of the ARC^3^Fusion components.

Module	Function and Necessity	Main Limitation
PSE	Constructs comparable source-aware features required for subsequent cross-modal relation modeling.	Computational cost scales with input resolution.
MSRA	Recovers modality-sensitive responses that may be attenuated by shared feature encoding.	Adds lightweight residual adaptation branches.
CMERD	Separates consensus from candidate residuals and provides the representations required by subsequent relation modeling.	Uses task-driven soft decomposition.
TCV	Verifies candidate residuals before fusion using source-aware and frequency-pattern evidence.	Relies on learned verification cues.
CMCE	Identifies local incompatibility between verified modality-specific responses.	Conflict is estimated from feature cues.
CAR	Converts consensus, reliability, and conflict evidence into adaptive fusion weights.	Adds spatial routing operations.
Decoder	Reconstructs the fused luminance image from the routed representation.	Produces luminance information only.
